# Reliability of Neonatal Screening Results

**DOI:** 10.3390/ijns4030028

**Published:** 2018-09-06

**Authors:** Maria Knapkova, Kate Hall, Gerard Loeber

**Affiliations:** 1 Newborn Screening Center SK, Children’s. Faculty Hospital. Banská Bystrica 97401, Slovakia; 2 The Spinney, Sutton Coldfield, B75 5NQ, UK; 3 ISNS office

## 1. Introduction

This special edition of the International Journal of Neonatal Screening comprises the abstracts of all oral presentations and posters from the biennial ISNS European regional meeting, to be held in Bratislava, Slovakia, 14–17 October 2018.

While some neonatal screening programs around the globe have run for more than fifty years, scientific and technological advancements are rapidly enabling neonatal screening for ever more congenital conditions to become available. This potential to significantly expand the scope of neonatal screening reminds us that it is worthwhile reviewing experience acquired by earlier neonatal screening programs and asking if the screening result is *reliable* i.e., to what extent does the result provide a true answer to the question of whether the infant suffers from the condition sought?

Factors involved in setting up a reliable screening system include establishing a clear definition of the disorder, determining which analytical parameters in body fluids are abnormal and can be analyzed with sufficient analytical precision to allow concentration or value ranges to be set to indicate a normal or a suspicious result with minimal overlap. Whether the infant’s gender, ethnicity, gestational age at birth, age at sampling or nutritional status should be considered in interpreting the result is very important.

The conference emphasizes the need to understand the reliability of screening results based on reflections on long-term experiences of screening for certain conditions such as congenital hypothyroidism. Time is also devoted to developments for two relatively new conditions, i.e., severe combined immunodeficiency (SCID) and spinal muscular dystrophy (SMA). Finally, the current neonatal screening situation in Europe is highlighted.

## 2. Invited Presentations

### I1. Some Notes on the History of Newborn Screening in Slovakia

DluholuckýSvetozárNewborn Screening Center SK, Children’s, Faculty Hospital, Banská Bystrica 97401, Slovakia

Newborn Screening (NS) in Slovakia started in 1985, after a six-year pilot study, testing logistics and collaborating individual healthcare workplaces. Screening of congenital hypothyroidism (CH) was performed by a T4 RIA suite with a thyroid stimulating hormone (TSH) confirmation, later exchanged for TSH ILMA with T4 confirmation. Screening of PKU/HPA was associated at one center following the introduction of the phenylalanine dried blood spot (DBS) (1995) fluorometric assay. Further screening of congenital adrenal hyperplasia (CAH) by 17OHP ILMA was introduced by r. 2003, cystic fibrosis (CF) (IRT) screening in 2009. Screening of selected inherited metabolic diseases (IMD) MS/MS in 2012 and another 10 IMDs in pilot mode were added in 2013. At the moment, there are 23 failures in a single screening center in Slovakia. A specific feature of Slovak Screening is its coding system that allows the child identification by birthplace, the identification of escape from screening, and the number of examined children at the given time. The system allows to follow-up the screening population coverage, which is permanently almost 100%. Another specific point is that the second recall of suspected cases is provided at regional recall centers that assure further care in confirmed cases at the level of children’s university hospitals. Continuous exchange of information between the screening center and recall centers allows monitoring of the actual incidence of malfunctions and possible correction of diagnosis. Since the introduction of NSF CF, it was necessary to start a follow-up of newborns according to ethnicity, as Roma children had a significantly higher cut-off value of both IRT1 and IRT2. This tracking allowed us to assess the ethnic incidence of all types of malfunctions. Since the start of NS in Slovakia, we have noted several good moments: The cumulative incidence of CH rises steadily over the years. While in 1985–1990 it was 1:5300, it was up to 1:1700 in 2017. The incidence of CH is higher in the Roma ethnicity (Index 1.3), its share in the rise of the secular incidence is not significant. The incidence of PKU/HPA has not changed over the years, there are no ethnic differences. Current MS/MS screening sensitively detects HPA cases in immature newborns, depending on nutrition. The incidence of CAH has a decreasing trend, probably due to a reduction in false positivity in the perinatal period (e.g., PICU patients). In Roma ethnicity the incidence of CAH is exceptional and its incidence is close to zero. Similarly to CAH, even in CF screening, although Roma have a 30% higher IRT, cystic fibrosis has been detected and confirmed in only one case of the Roma neonates. NS CF does not detect milder forms of cystic fibrosis. IMD screening using MS/MS revealed significant ethnic differences in incidence and spectrum of disorders. While the incidence of IMD in the majority population is 1:1880, in the Roma population even 1:121. The IMD spectrum in the majority population is dominated by PKU, medium-chain acyl-CoA dehydrogenase deficiency (MCAD), in the Roma population, it is MCAD, SCAD, CUD. In the large SCAD file, two new alleles of gene mutations were discovered, one of which had a potential lethal course (2 cases of SIDS), were found exclusively in Roma children. These findings are the subject of further studies. The ethnical approach to screening brings, in addition to focused care, new insights into this autochthonous minority and its origin.

### I2. NBS Is Pandora’s Box; New Techniques Make Anything Possible

BonhamJim R.Sheffield Children’s Hospital, Sheffield S10 2TH, UK

There is little doubt that the advent of population-based newborn screening has benefitted many patients and families since its adoption in the 1960s. Indeed, a meeting held in Atlanta in 2012 to celebrate 50 years of newborn screening suggested that the lives of 12,000 babies per year are being saved or improved through this intervention in the US alone and the expansion in the number of conditions tested and the countries who choose to adopt these programs continues to increase throughout the world. When first described the detection of phenylketonuria depended upon a specific test to identify an increased concentration of phenylalanine in neonatal blood. The subsequent addition of screening for congenital hypothyroidism in the 1980s in many countries similarly depended upon a specifically designed test for a signature compound by a distinct technique. The introduction of enzyme linked immunoassays allowed generic approaches albeit with a single assay for each disorder. Electrospray injection tandem mass spectrometry in the 1990s changed this landscape by offering an approach to detect a range of up to 30 conditions using a single inexpensive test where a suitable key biochemical metabolite could be identified. More recently the introduction of DNA-based techniques such as T-cell receptor excision circle (TREC) analysis and next generation sequencing capable of using a dried blood spot sample provides the basis to extend the range of conditions to include those such as SCID and fragile X where no suitable biochemical marker exists. Biochemical, both metabolite and enzymic, and genetic testing combined with “big data” analysis to identify key environmental factors related to childhood well-being and development could go further to provide a comprehensive approach to improve the life chances of many children. Nevertheless, like Pandora’s Box there can be serious and unforeseen side effects from the reasonable wish to identify and help those at risk. The weight attached to these “disutilities” when planning public health interventions varies depending upon those making the decisions. Typically, clinicians involved with treating those affected favor the early detection offered by screening whereas public health planners are somewhat more guarded, attempting to balance the risks for those who may be medicalized unnecessarily compared with the benefits to the small number who can be offered early treatment. An intelligent discussion of the decision around the introduction of new screening programs should perhaps not simply seek to establish the benefits of screening but also address how key disutilities may be ameliorated. These include technical considerations such as improved means of assigning screen positive results and secondary testing but also a qualitative understanding of how public engagement, pre-screening information and the careful delivery of screen positive results can lessen the negative impact of screening programs for the families affected. Closer attention to case definition at the outset with agreed and universally applied diagnostic protocols may do much to reduce uncertainty and facilitate meaningful outcome studies. Such clarity of definition and forward planning should perhaps be a pre-requisite of any new program development. By this means we may be able to assess the longer-term effects from lifting the lid on Pandora’s Box and understand more clearly how to limit some of the harms resulting from screening while preserving the benefits that are so important to patients and families.

### I3. Does It Count? Defining Disorders

WileyVeronicaNSW Newborn Screening Program, Sydney Children’s Hospital Network, Wentworthville, NSW 2145, Australia

Assessing the efficacy of a population screening program requires evaluation of the entire system including the pre-analytical, analytical and post-analytical aspects. While this has been common practice for over 50 years since the World Health Organization commissioned Wilson and Jungner to develop “Principles and Practice of Screening for Disease” [1], there remains a lack of national and international agreement on what should be included in newborn screening programs and what should be counted. Many of the conditions under current consideration as a pilot program of either a country, or at least region within a country, (for example: lysosomal storage disorders and neuromuscular disorders) have diverse clinical symptoms ranging from acute neonatal presentation to those with presentation late in childhood or adulthood, if at all. However, this is not really a new phenomenon with most disorders included in newborn population screening having mild or late-onset forms including phenylketonuria, hypothyroidism, cystic fibrosis and congenital adrenal hyperplasia. Carefully defining what is being sought is necessary to appropriately assess effectiveness. What constitutes a positive result must be defined for the determination of sensitivity (affected persons with a positive result/all affected); specificity (healthy persons with a negative result/all non-affected); and positive predictive value (proportion with a positive result that are affected). In Australasia, the newborn screening committee with clinical and scientific representatives from professional societies advising on aspects of newborn bloodspot screening including the Human Genetics Society of Australasia (HGSA), and the Australasian College of Physicians have developed definitions of disorders for data gathering and to ensure comparability of data. The definitions currently available on the HGSA website recognized that for all cases there needs to be diagnostic evidence and that case finding remains a continuing process and not a one-time project.

1. Wilson, J.M.G.; Jungner, G. Principles and Practice of Screening for Disease. Public Health Papers 1968. Available online: http://apps.who.int/iris/bitstream/10665/208882/1/WHO_PA_66.7_eng.pdf (accessed on 15 June 2018).
2. HGSA (Human Genetics Society of Australasia) Policies. https://www.hgsa.org.au/resources/hgsa-policies-and-position-statements (accessed on 15 June 2018).

### 2.4. Long-Term Outcome Studies, the (Often) Neglected Part of Newborn Screening Programs

KölkerStefanDivision of Pediatric Neurology and Metabolic Medicine, Center for Pediatric and Adolescent Medicine, University Hospital Heidelberg, Heidelberg 69120, Germany

Newborn screening (NBS) programs are intended to identify newborns with treatable conditions and to enable the implementation of a therapy before patients suffer irreversible damage. Their goal is thus to improve health outcomes and quality of life of affected individuals. For many diseases, however, the impact of NBS has remained unclear for various reasons. Long-term observational studies are valid tools to overcome these uncertainties and to elucidate whether NBS programs achieve their goal. This is discussed for glutaric aciduria type 1 (GA1), isovaleric aciduria (IVA), (isolated) methylmalonic acidurias (MMA) and propionic aciduria (PA). While GA1 and IVA have been included into an increasing number of NBS programs worldwide, there is still reservation towards inclusion of MMA and PA. When NBS pilot studies for GA1 were initiated two decades ago, evidence was weak that this condition was treatable at all. Meanwhile, studies in different countries confirmed that the neurologic outcome was significantly improved by NBS. However, the patient benefit critically relied on the therapeutic quality: both non-adherence to recommended emergency treatment and low lysine diet clearly increased the risk of striatal damage. Regardless of NBS and therapy, however, chronic kidney disease develops over time in some patients, while loss of GCDH activity was associated with progressing white matter abnormalities of unclear clinical relevance. In contrast to GA1, the benefit for IVA patients is less clear. In comparison to other organic acidurias IVA is exceptional considering its milder neurologic phenotype and the fact that NBS also identifies individuals with mild IVA, a benign disease variant. Although NBS seems to reduce neonatal mortality and to improve neurologic outcome, this positive effect was not confirmed by a recent European study after exclusion of individuals with mild IVA. To avoid overestimation of NBS-related benefits, the case mixes of NBS and pre-screening cohorts must be carefully balanced. MMA and PA are not implemented in the majority of NBS programs since the specificity of C3-based NBS is low and patients with neonatal disease onset may not benefit from NBS. A recent European study showed that about 60–65% of MMA and PA patients remained asymptomatic during the first week of life. Cobalamin-nonresponsive MMA patients identified by NBS were less likely to develop motor abnormalities, while screened PA patients had a lower risk for cardiac manifestation with age than those not screened. With the availability of two or multiple tier NBS technologies these results may encourage to reconsider the inclusion of MMA and PA into NBS programs. These examples show that observational studies are a powerful tool to elucidate the long-term benefits of patients identified by NBS and to optimize and harmonize therapy and management.

### I5. Neonatal Screening for Congenital Hypothyroidism: 50 Years of Changing Definitions

TorresaniToni8132 Egg, Switzerland

Neonatal Screening is a public health initiative, which has been implemented for more than 50 years. Unfortunately, quite often the fact that a neonatal screening test simply indicates that a baby might have a condition, is overlooked. Furthermore, when different programs compare their performances, the different conditions for sample collection, transport and analysis are not taken into consideration. Whether a neonatal screening result can be classified as within or outside limit, is not only dependent from the above-mentioned conditions, but also from several clinical conditions such as maturity, time of sample collection, general health condition of the baby and sometimes also from the mother. The above-mentioned factors have contributed to a change in the definition of the aims of neonatal screening for congenital hypothyroidism. Numerous contributions and publications have appeared in recent years covering this topic. A selected overview of the relevant literature will be presented and discussed.

### I6. Detection of Congenital Adrenal Hyperplasia: Is There a Need to Screen All Babies Twice?

HeldPatrice K.^1^*ShapiraStuart K.^2^HintonCynthia F.^2^JonesElizabeth^3^HannonW. Harry^4^OjoduJelili^3^1Wisconsin State Laboratory of Hygiene, University of Wisconsin, Madison, WI 53706, USA2National Center on Birth Defects and Developmental Disabilities, Centers for Disease Control and Prevention, Atlanta, GA 30341, USA3Newborn Screening and Genetics Program, Association of Public Health Laboratories, Silver Spring, MD 20910, USA4Division of Laboratory Sciences, Centers for Disease Control and Prevention (retired), Atlanta, GA 30341, USA*Corresponding author

There is no clear consensus among state newborn screening programs on whether routine second screening of newborns identifies clinically relevant cases of congenital adrenal hyperplasia. This retrospective study evaluated laboratory practices, along with biochemical and medical characteristics of congenital adrenal hyperplasia (CAH) cases (1) detected on the first newborn screen in one-screen compared to two-screen states, and (2) detected on the first versus the second screen in the two-screen states, to determine the effectiveness of a second screen. A total of 374 confirmed cases of CAH from 2 one-screen states and 5 two-screen states were included in this study. Demographic data and diagnostic information on each reported case were collected and analyzed. Additionally, laboratory data, including screening methodologies and algorithms, were evaluated. The one-screen states reported 99 cases of CAH out of 1,740,586 (1 in 17,500) newborns screened: 88 (89%) identified on first screen and 5 (5%) identified on targeted second screen. The two-screen states reported 275 cases of CAH out of 2,629,627 (1 in 9500) newborns screened: 165 (60%) identified on first screen and 99 (36%) identified on second screen. Using a multivariate model, the only significant predictor of whether a case was identified on the first or second screen in the two-screen states was the type of CAH. Compared with classical salt-wasting CAH, classical simple virilizing and non-classical CAH cases were less likely to be detected on the first versus the second screen. The routine second newborn screen is important for identifying children with CAH, particularly simple virilizing and non-classical forms, which might otherwise not be captured through a single screen. Mol Genet Metab. 2015 November; 116(3): 133–138.

### I7. Precision Newborn Screening Driven by Results Adjustments for Multiple Covariates

RinaldoPieroBiochemical Genetics Laboratory, Department of Laboratory Medicine and Pathology, Mayo Clinic, Rochester, MN 55905, USA

Virtually all medical specialties are challenged by utilization management and precision medicine forces, so public health and specifically the performance of newborn screening are unlikely to be exempted from the same scrutiny. Newborn screening (NBS) is based upon laboratory tests performed on a growing proportion of ~130 million children born worldwide every year. Poor performance on a mass scale distresses a multitude of patients, and exposes both families and providers to an increasing risk of psychosocial harm while incurring in unnecessary expenses. Our multidisciplinary team is focused on the creation of high-throughput post-analytical interpretive tools to improve NBS performance. Our goal is to achieve a near-zero false positive rate (FPR), which is the proposed definition of precision newborn screening. Collaborative Laboratory Integrated Reports (CLIR 2.09; https://clir.mayo.edu) is a second-generation web application that maintains an interactive database of laboratory results from multiple sites. The CLIR tools are applicable to either the diagnosis of one condition or to the differential diagnosis between two conditions with overlapping phenotypes (affected vs. heterozygote; true positives vs. false positives). CLIR’s defining characteristics are the replacement of analyte cut-off values with condition-specific degree of overlap between cumulative reference and disease ranges, and the integration of primary markers with all informative permutations of ratios. Ratios calculated between markers not directly related at the biochemical level are particularly helpful to correct for pre-analytical factors and potential analytical bias. An additional and unique feature of CLIR is the replacement of conventional reference intervals with continuous, covariate-adjusted (age, birth weight, sex) moving percentiles. Harmonization by location is also routinely possible. Access to CLIR is freely available to qualified users worldwide willing to share reference data and profiles of positive cases in advance of being given access to the website. The goal of collaboration and data sharing is to sustain a constantly evolving, and improving, clinical validation. The type of statistical modeling that takes place within CLIR requires big data, and indeed a willingness to evaluate the concept that reference intervals could be defined by “recycling” and harmonizing vast amounts (>>1 M) of normal screening test results from a multitude of sources. As an example, over a two-year period (N = 116,469) we have achieved an FPR of 0.0009% and a positive predictive value (PPV) of 87% for NBS of three lysosomal disorders. FPR reduction for non-MS/MS conditions between 50% and 80% is also achievable.

### I8. Newborn Screening for Severe Combined Immunodeficiency (SCID)

GasparBobbyProfessor of Pediatrics and Immunology, UCL Great Ormond Street Institute of Child Health, 30 Guilford Street, London WC1N 1EH, UK

Severe combined immunodeficiency (SCID) is the most severe form of inherited primary immunodeficiency and is a pediatric emergency. Delay in recognizing and detecting SCID can have fatal consequences and reduces the chances of a successful hematopoietic stem cell transplant (HSCT). Screening for SCID at birth would prevent children from dying before HSCT can be attempted and would increase the success of HSCT. There is strong evidence to show that SCID fulfills the internationally-established criteria for a condition to be screened for at birth. There is also a test (the T-cell receptor excision circle (TREC) assay) that is now being successfully used in an increasing number of US states to screen for SCID in routine newborn Guthrie samples. Concerted lobbying efforts have highlighted the need for newborn screening (NBS) for SCID, and its implementation is being discussed in Europe both at EU and individual country level, but as yet there is no global mandate to screen for this rare and frequently lethal condition. This session will summarize the current evidence for, and the success of SCID NBS, together with a review of the practical aspects of SCID testing and the arguments in favor of adding SCID to the conditions screened for at birth.

### I9. Newborn Screening for Severe Combined Immunodeficiencies–IPOPI Perspective

SolisLeireHealth Policy and Advocacy Manager, International Patient Organization for Primary Immunodeficiencies (IPOPI), Estoril 2765-187, Portugal

Newborn screening for rare diseases has been a successful method of speeding up diagnosis and treatment of diseases. In case of diseases in need of a timely diagnosis and early access to treatment, such as severe combined immunodeficiencies, newborn screening is of paramount importance and should be seen as a pediatric emergency. Screening newborns to identify whether they could have SCID is possible and is already ongoing in some countries and regions of the world. It is already happening and helping patients access, once the diagnosis is confirmed, their life-saving curative treatment. From a patient perspective and given the availability of curative treatments (HSCT and gene therapies), every country should take the necessary steps to ensure that the lives of children with SCID can be saved. The first crucial step to do this is to screen all newborns for SCID. IPOPI, as the International Patient Organization for Primary Immunodeficiencies, has been supporting initiatives and programs aimed at achieving the implementation of SCID newborn screening. We are working at European level and supporting national campaigns of some of our members to ensure a timely implementation. From IPOPI’s perspective, only by establishing a partnership between healthcare professionals, screening experts and patients, can advocacy campaigns be successful in making SCID newborn screening a reality that will help avoiding the loss of many babies’ lives.

### I10. Newborn Screening for SCID in New Zealand

D.WebsterM.de HoraP.DrylandJ.SinclairK.HsiaoS.BrothersNational Testing Center, Auckland AK1, New Zealand

Adding disorders to the New Zealand newborn screening program follows a well-documented process and this was followed for the addition of SCID including a cost utility study which showed the Quality Adjusted Life Year (QALY) cost similar to other healthcare interventions and screening started in December 2017. Although the Policy Framework lists introduction of new screening tests as a primary use of the dried blood spot sample the Ministry of Health National Screening Unit (NSU) did not approve use of anonymized samples for assay set up and validation and a protocol was agreed whereby following the Ministerial announcement that screening would start a limited number of named samples could be used with any suspicious results reviewed using the final assay protocol and families contacted if appropriate (fortunately none found). An implementation group with membership from the NSU, laboratory and the pediatric immunology team was formed and selected the Perkin-Elmer Enlite Neonatal TREC kit due to a tight timeframe for implementation. Information for families and others was developed and along with other resources is publicly available. Technical issues identified have been kit fragility with increased temperature in shipping; microtiter plate sealing and PCR instruments. Due to the small but geographically dispersed population there have been some difficulties getting the appropriate diagnostic samples to the central testing laboratory in a timely way. The suggested kit cut-offs on screening data would have given a high recall rate so these have been, and are, under review. The agreed follow-up pathway of clinical referral when disease is likely has produced the expected number of positive results (6 in about 30,000) of whom none have the condition. Policy framework https://www.nsu.govt.nz/system/files/page/newborn_metabolic_screening_programme_policy_framework_june_2011.pdf. SCID FAQs and cost utility study etc. https://www.nsu.govt.nz/health-professionals/newborn-metabolic-screening-programme/screening-severe-combined-immune. Information resources https://www.nsu.govt.nz/pregnancy-newborn-screening/newborn-metabolic-screening-programme-heel-prick-test/information.

### I11. Newborn Screening for SCID-The Dutch Approach

SchielenPeter^1^BlomMaartje^1^DekkersEugenie^2^BrediusRobbert^3^BurgMirjam van der^4^Groupon behalf of the SONNET Study1Reference Laboratory for Neonatal Screening, Center for Health Protection Research, Dutch Institute of Public Health and the Environment (RIVM), 37210 BA Bilthoven, The Netherlands2Center for Population Screening, RIVM, 3720BA Bilthoven, The Netherlands3Department of Pediatrics, Leiden University Medical Center, 2333ZA Leiden, The Netherlands4Department of Pediatrics, Laboratory for Immunology, Leiden University Medical Center, 2333ZA Leiden, The Netherlands

In July 2015 the Dutch Minister of Health sanctioned an advice of the Dutch Health Council to expand the newborn screening program with screening for 17 diseases, with special reference to one disease in particular; severe combined immunodeficiency (SCID). While for all other diseases, the Center for Population Screening of the National Institute for Public Health and the Environment (RIVM-CVB) was invited to perform a feasibility study, for SCID screening RIVM-CVB was asked to enable a prospective pilot screening to identify and overcome all challenges of the introduction of SCID screening in the routine screening program. Thus, a project group was composed with representatives of all relevant fields of expertise, including clinical genetics, clinical chemistry, medical ethics, immunological pediatrics and the screening process and policy. This group composed a study proposal to screen 70,000 neonates, nested in the routine screening program. The proposal consisted of four work packages, covering (1) all organizational aspects of the pilot screening (literally from the heel of the neonate to screening result), (2) the actual pilot screening of 70,000 neonates, including the evaluation of the screening policy, (3) a concise cost-effectiveness analysis and (4) ethical, legal and social implications. In this presentation, within the frame of this potentially typically Dutch approach, some of the challenges of the pilot screening are highlighted (for instance SCID case definition, the choice of the TREC screening test, and choices for the diagnostic follow-up). Secondly, as the pilot screening started 3 April 2018, first results are presented in light of the theme of this meeting; ‘Reliability of screening results’.

### I12. SCID Screening: A Public Health Perspective

HailstoneSimonUK National Screening Committee, 133-155 Waterloo Road, London SE1 8UG, UK

In November 2017 the UK National Screening Committee recommended that SCID screening was evaluated in the UK. Implementing this recommendation has presented numerous challenges to the project steering group and has raised important questions about how new tests are incorporated into the current new born screening system. Balancing the likely benefits of screening for this condition against the numerous feasibility issues has been challenging but the approach being developed is likely to have significant benefits in the future as new screening tests are developed and ultimately become more common-place in the genomics era. This presentation gives a public health perspective on the UK experience of developing a SCID-screening evaluation and how reliable the findings of that evaluation may be given the scope and resources available.

### I13. Neonatal Screening for Severe Primary Immunodeficiencies in Sweden

ZetterströmRolf H.^1^,^2^OhlssonAnnika^1^,^3^JonssonSusanne^1^BarbaroMichela^1^,^2^BorteStephan^4^,^5^WiniarskiJacek^6^,^7^HammarströmLennart^4^DöbelnUlrika von^1^,^3^1Center for Inherited Metabolic Diseases, Karolinska University Hospital Solna, SE-17176 Stockholm, Sweden2Department of Molecular Medicine and Surgery, Karolinska Institutet, SE-17176 Stockholm, Sweden3Department of Medical Biochemistry and Biophysics, Division of Molecular Metabolism, Karolinska Institutet, SE-17177 Stockholm, Sweden4Department of Clinical Immunology, Karolinska University Hospital Huddinge, SE-14186 Stockholm, Sweden5ImmunoDeficiencyCenter Leipzig (IDCL) at Hospital St. Georg Leipzig, Delitzscher Strasse 141, 04129 Leipzig, Germany6Department of Clinical Technology and Intervention, Karolinska Institutet, SE-14186 Stockholm, Sweden7Department of Pediatrics, Karolinska University Hospital Huddinge, SE-14186 Stockholm, Sweden

In Sweden screening for phenylketonuria was initiated 1965. The latest extension of disorders was in 2010 when 19 new diseases were added to the screening program making the total number of disorders 24. Since 2014 new disorders are added after a process at the Swedish Board of health and Welfare based on the Wilson and Jungner criteria. Adding severe combined immunodeficiency (SCID) to the program is currently under review. Between November 2013 and November 2016, we performed a pilot study in Stockholm County using a real-time polymerase chain reaction triple assay measuring T-cell receptor excision circles (TRECs), Kappa-deleting recombination excision circles (KRECs) and beta-actin simultaneously. 89,462 newborns were tested and five were found to have primary immunodeficiencies. Two had a combined immunodeficiency with unknown genetic cause, one had ataxia telangiectasia and two had true SCID; one with Artemis deficiency and the other with adenosine deaminase deficiency. No child with Bruton’s agammaglobulinemia was found during the study period but 19 children with low KRECs were identified all with mothers on immunosuppressive treatment during pregnancy. The numbers of KRECs normalized in all these children. Since the triple assay used during the study period is not optimal for a nationwide SCID screening in Sweden with approximately 115,000 newborns per year we recently performed a comparative study using two commercially available assays. The results from this study will also be presented.

### I14. Needs and Perspectives of Future Neonatal Screenings for Severe Immunodeficiencies

BorteStephanKarolinska University Hospital Huddinge, Stockholm S141868, Sweden

Primary immunodeficiency diseases (PID) have paved their way from research-level projects into a growing number of national screening programs, recently. Characterized by inborn profound T-cell lymphopenia, severe combined immunodeficiency (SCID) has been the first PID to become part of a growing number of new disease entities recognized as a relevant condition with curable treatment options to be identified at the neonatal stage, before mortality and morbidity deteriorates patient’s life. However, a multitude of technological, logistical and ethical challenges have limited the impact and thrive of screening approached ‘beyond SCID’ to come into action. The needs and perspectives of future newborn screening programs for other PID will be exemplified and discussed.

### I15. How Does TSH Cut-Off Impact the Reliability of Congenital Hypothyroid Screening?

HeatherNatashaAuckland City Hospital, Grafton, Auckland AK1, New Zealand

Newborn screening thyroid stimulating hormone (TSH) cut-offs vary widely throughout the world. This can partly be explained by physiological factors such as age at sample collection, demographic factors such as ethnicity and variation in analytical values produced by kits from different manufacturers. The potential impact of these factors and significance to screening programs will be discussed. Although some screening programs adjust cut-offs following cases missed when levels are below the limit, this approach does not necessarily improve overall screening parameters e.g., positive predictive value. Since 1986 New Zealand screening for congenital hypothyroidism has used a primary TSH test with a cut-off of 15 mIU/L blood. Within our local region the New Zealand cut-off is relatively high, and we sought to determine whether it was too high. An audit of missed cases found those missed due to immaturity, secondary hypothyroidism, twin-twin transfusion and TSH levels below screening cut-off. In addition, we systematically searched for missed cases and neuro-cognitive impairment among children with newborn screening TSH levels just below cut-off (subclinical hypothyroidism, SCHT, TSH levels 9–14 mIU/L blood). 90 children aged 6–11 years with untreated SCHT at birth and 76 sibling controls were recruited. The SCHT group underwent thyroid function tests and all underwent comprehensive cognitive assessment. Provisional results are of no missed cases of congenital hypothyroidism and that neuro-cognitive outcomes do not differ between the SCHT and sibling control groups.

### I16. The Spectrum of Phenotypes in Children with Congenital Hypothyroidism in West Slovakia

NogeovaAdreanaA. Getlik Clinic for Children and Adolescents SMU, Faculty of Medicine, 036 59 Martin, Bratislava, Slovakia

According to Newborn Screening Center of Slovak Republic (NSC SR) Annual report of 2017, between 1985 and 2017 in congenital hypothyroidism (CH) screening, 2,041,784 newborns were screened, and 594 thyroid disorders were detected. The incidence of the disease is 1:3437 births. The spectrum of phenotypes in children with congenital hypothyroidism in West Slovakia includes a broad spectrum of thyroid dysfunctions with heterogeneous etiologies: most patients have a primary hypothyroidism due to thyroid aplasia and hypoplasia, sublingual and retrosternal ectopia of the thyroid gland and dyshormogenesis, i.e., disturbance of biosynthesis or thyroid hormone transport. Two patients have central hypothyroidism with consequent disorder of thyroid gland development. In all these patients an impaired thyroid hormone production is permanent. In our region there are also some patients with transient forms of primary congenital hypothyroidism with normal thyroid function tests after trial-off therapy in the age of 3 years. Substitution treatment in most of our patients is thanks to excellent collaboration with the Screening Center of Slovakia initiated up to 9–10 days after birth.

### I17. Critical Evaluation of the Dutch Neonatal Screening on Central Congenital Hypothyroidism

StroekKevin^1^SchielenPeter^2^BoelenAnita^1^1Neonatal Screening Laboratory Amsterdam, Laboratory of Endocrinology, Academic Medical Center, Meibergdreef 9, 1105 AZ Amsterdam, The Netherlands2Laboratory for Neonatal Screening, Center for Health Protection, National Institute for Public Health and the Environment, NL-3720 BA Bilthoven, The Netherlands

Congenital hypothyroidism (CH) comprises a variety of disorders of either the thyroid gland (CH-T) itself or the regulatory system stimulating the thyroid gland consisting of the hypothalamus and the pituitary gland (central congenital hypothyroidism, CH-C), which both result in a lack of thyroid hormone (TH) in the neonatal period. TH is essential for brain development and for this reason, a neonatal screening program for CH was established in the Netherlands in 1981. Although many screening programs are based on thyroid stimulating hormone (TSH), the Dutch neonatal screening scheme for CH is primarily based on the determination of thyroxine (T4) in filter paper blood spots. The concentration of T4 is expressed as a standard deviation (T4SD) score of the daily mean. In the lowest 20% of T4 values, TSH concentrations are measured additionally. Since 1995, in the lowest 5% of T4 values, thyroxine binding globulin (TBG) is measured and a calculated T4/TBG ratio then serves as indirect measure of the FT4 concentration. This ratio is used to exclude neonates with TBG deficiency leaving neonates with potential CH-C. This screening program detects congenital hypothyroidism of varying severity and etiology in a cost-effective way. However, increasing reports of false positive cases of CH-C require that we critically evaluate the current program as false positive results are known to cause psychological problems in parents and altered perceptions of the health of their child. To this end, we analyzed a data set of 2492 referred cases in the period 2007–2015 (premature babies excluded) and a data set of 1234 referred cases in the period 2012–2015 (premature babies excluded). The latter set is used to analyze the specificity of CH-C as the Dutch laboratories switched in 2012 to a non-radioactive TBG immunoassay. The diagnosis CH-C is established in only 2.4% of the children (17 cases) who were referred based on two consecutive abnormal T4/TBG ratios (≤17), with a high number of false positives (691) indicating a poor specificity of the screening for CH-C. Lowering the T4/TBG ratio cut-off value in the second heel prick to 16, 15, 14, 13 and 12 respectively results in a reduction of 176, 358, 483, 578 and 646 false positives, at the cost of an increase in false negatives of 2, 3, 4, 4 and 5. Lowering the T4/TBG ratio cut-off value in both the first and the second heel prick results in markedly higher numbers of false negatives. Unlike the screening on CH-C, a high percentage (91%) of children with a positive TSH value (≥22 mIU/L whole blood) does indeed have CH-T, indicating an excellent specificity of this part of the screening program. The present study evaluates the specificity of the CH-C screening, part of the Dutch Neonatal Screening Program. The screening on CH-C is mainly based on the determination of the T4/TBG ratio as an indirect measure of fT4 concentrations. Although the screening on CH-C is proven to be cost-effective, the specificity is poor, especially since the laboratories switched to an alternative TBG assay. Lowering the cut-off value for the ratio significantly lowers the number of referrals while the increase of missed cases is limited. Thus, adaptation of the cut-off value of the T4/TBG ratio is the first step to improve the specificity of the neonatal screening on CH-C. 

### I18. Cut-Off Determinations and Risk Assessment Methods in Dried Blood Spot Newborn Screening

MeiJoanneOrsiniJoeHuntPatriciaZarbalianGuisouPetritisKostasOjoduJeliliMembers of the Association of Public Health Laboratories (APHL)Newborn Screening QA/QC Subcommittee and APHL Newborn Screening and Genetics in Public Health Committee, Silver Spring, MD 20910, USA

Newborn screening (NBS) laboratories use a variety of approaches to determine if a newborn is at risk for a screened disorder. Newborns identified with abnormal biomarker levels indicate the need for additional testing. APHL organized a process to summarize approaches to establish and evaluate risk assessment methods. Cut-off values may be determined by: (1) conducting a population study to test dried blood spot specimens (DBS) from unaffected newborns; (2) analyzing data to determine whether the screen has adequate precision and accuracy; (3) assigning a preliminary cut-off value; (4) validating or verifying the cut-off using known positive control samples, preferably from residual DBS when available, and; (5) comparing cut-off values to other programs or databases. Cut-offs and risk assessment methods should be monitored and adjusted based on feedback from short- and long-term follow-up and clinical outcomes of infants, when new information about the disorder becomes available or when significant changes are made in the testing process that may affect the reference range. Borderline cut-offs are helpful in determining the need for a second specimen request or for biomarkers that fluctuate with gestational age or other factors. Fixed cut-offs or multiple of the median work for methods that directly measure biomarkers, while floating cut-offs are appropriate for functional assays. Online tools can be used to compare results from positive cases to marker distributions for affected populations. These approaches may be useful for developing or expanding NBS programs. NBS can identify newborns at higher risk, but may not detect all affected newborns. Algorithms may use multiple methods to balance the benefits and harms of screening.

### I19. Optimizing Pre-Analytical Processes to Ensure the Reliability of Newborn Screening Results

McRobertsChristineChildren’s Hospital for Eastern Ontario, Ottawa, ON K0E 1W0, Canada

Newborn Screening Ontario (NSO) continuously strives to deliver timely, efficient and cost-effective newborn screening (NBS) services and is making improvements to pre-analytical processes to maintain the analytical and clinical reliability of screening results. NSO has cultivated a co-operative, mutually beneficial relationship with healthcare providers (HCP) involved in dried blood spot (DBS) collection and transport. To support timely transport of quality DBS specimens, NSO offers e-learning and onsite workshops, a quarterly report providing site-specific performance feedback for NSO key performance indicators (KPI), and program-maintained specimen shipping and tracking tools. HCP are assisted in meeting KPI benchmarks by accessing NSO education materials intended to guide improvements to practice. Cargo, an internally developed package tracking system, supports reduced specimen transit times by providing early identification of delayed or lost packages. Cargo has successfully identified overdue and missing packages triggering earlier follow-up investigation for impacted specimens. NSO has partnered with STACS DNA to further improve the capability of the tracking system, implementing Track-Kit™, a web-based system allowing NSO to monitor the status of shipments at a specimen level. This web-based solution integrates with existing shipping workflows, linking to the online courier system to create shipping requests and waybills and eliminates manual tracking logs. Other benefits include better blood collection device inventory control and expiration warnings and improved ability to determine accurate specimen transport metrics. Working with HCP promotes cohesiveness and confidence in the Ontario screening system helping to ensure reliable screening results.

### I20. Screening for Neuromuscular Disorders. Long History, Novel Opportunities

KasperDavid^1^,^2^1ARCHIMED Life Science GmbH (ARCHIMEDlife), 1110 Vienna, Austria2Medical University of Vienna, Clinical Institute of Laboratory Medicine, 1220 Vienna, Austrai

The newborn screening (NBS) for neuromuscular disorders became of interest more than 40 years ago with the start of first Duchenne muscular dystrophy (DMD) programs. Even though several DMD programs were implemented none finally remained. Another lesson we have learned is from Pompe disease with the availability of a first enzyme replacement therapy since 2006. Several (pilot) programs were started worldwide with different aspects and currently several states in the US as well as countries such as Taiwan have implemented Pompe disease in their NBS panel. Spinal muscular atrophy (SMA) showing overlapping clinical symptoms with Pompe disease was also several times considered for NBS programs but rejected. In 2016, a promising therapy become available and now may open the doors for further NBS activities again. Nonetheless, there is a gap between the lack of a SMA-NBS program and the clinical needed for a fast, reliable and early diagnosis thus disease-awareness campaigns may support SMA diagnostics until public NBS programs are available. We also show the first data from a study offering a simple dried blood spot test to reduce diagnostic barriers and may ease physicians and their patients’ access to timelier diagnostics that could help to confirm a clinical diagnosis. At present, several treatments mainly with special focus on genetic-based therapies are under investigation for a range of neuromuscular disorders, offering the potential for significant improvements in patient care and might foster more NSB activities worldwide.

### I21. Clinical Presentation of Spinal Muscular Atrophy

OkalovaKatarinaNeurological Pediatrics, Children’s Faculty Hospital, Banská Bystrica 97401, Slovakia

Spinal muscular atrophy (SMA) is an autosomal recessive inherited neuromuscular disease primarily affecting children. It is a leading genetic cause of infant mortality and the second most common autosomal recessive genetic disorder after cystic fibrosis. The disease affects 1 in every 6000 to 10,000 newborns. SMA is caused by homozygous mutations in the survival motor neuron 1 (SMN1) gene and retention of at least one copy of the homotrophic gene paralog SMN2.

SMA causes degeneration and loss of α motor neurons in the anterior horn of the spinal cord, leading to progressive muscle weakness and in severe cases respiratory failure and death.

SMA presents across a broad clinical spectrum in terms of age of onset and severity of disease and is classified into different types based on onset and highest motor function milestones achieved.

Patients with the type I form of SMA, which is also the most common form, show signs of the disease soon after birth (˂6 months of age), never gain the ability to sit and typically do not survive past 2 years of life. Patients with the intermediate form of the disease (type II) are affected before 18 months of age and can sit upright, but never gain ability to stand without support. A milder form of the disease (type III) begins after 18 months of age and patients maintain the ability to stand on their own and live a normal lifespan. Additional forms of SMA have been identified at both ends of severity spectrum: type 0 SMA is a prenatal form of the disease that is uniformly fatal, is presented in first days of life and type IV SMA is an adult onset form with mild proximal muscle weakness and normal life expectancy.

### I22. Spinal Muscular Atrophy: A Challenging Disease for Newborn Screening?

ServaisLaurent^1^DangouloffTamara^2^BoemerJean-Hubert Caberg Francois^1^1Pediatric Department, CHU de Liège, Liège 4000, Belgium2Genetic Department, CHU de Liège, Liège, 4000 Belgium

Spinal muscular atrophy (SMA) is a devastating motor neuron disease that affects approximately 1 in 10,000 newborns. It is caused by loss of function of the SMN1 gene. In 95% of cases, exon 7 of SMN1 is not included in the mature mRNA. In its most severe and frequent form (50–60% of cases), called SMA type 1, severe hypotonia appears during the first months of life, resulting in death within two years or severe disability. About 30% of cases are SMA type 2. These patients first present symptoms between 6 months and 18 months, acquire an independent sitting position, but never walk autonomously. SMA type 3 starts after the age of 18 months and is associated with loss of ambulation in about 50% of cases. All types are associated with high societal and familial economic burden. The annual cost of SMA treatment is estimated at 52.000 euros for type 3 and 107.000 euros for type 1. Homozygous null mutations in *SMN1* cause all types of SMA, but the number of copies of *SMN2*, a homologous hypofunctional gene present in all patients, accounts for the large phenotypic variation. The more copies of *SMN2*, the better the prognosis, although this correlation is not absolute. Recently, a phase 3 trial in type 1 patients demonstrated that Nusinersen, an intrathecally injected antisense oligonucleotide that modifies splicing of *SMN2* pre-mRNA promotes increased production of the full-length SMN protein, prolongs event-free survival, and allows patients to achieve new motor milestones. Nusinersen is now FDA- and EMA-approved and is reimbursed in the USA and several European countries to up to 300,000 euros per year. Simultaneously, a phase 1 study demonstrated the efficiency of *SMN1* gene transfer through self-complementary AAV9 vectors in a cohort of 15 type 1 patients. Both studies demonstrated that early treatment was associated with greater efficacy. Preliminary results indicate that patients treated with Nusinersen before the appearance of symptoms achieve a normal or nearly normal motor development with a therapeutic effect far above the one observed in post-symptomatically treated patients. These results have encouraged pilot studies of an NBS for SMA in New York (USA) and in Taiwan. Pilot programs are going to be initiated this year in Bavaria (Germany), Tuscany, Lazio (Italy), Catalonia (Spain), and Southern Belgium. In July 2017, Missouri (USA) introduced NBS for SMA into the law. On 8 February 2018, the Advisory Committee on Heritable Disorders in Newborns and Children recommended that newborn screening for SMA be implemented across the USA. The recommendation requires approval by the Health and Human Services Secretary. On March 5 of this year NBS for SMA using a PCR-based assay started in Southern Belgium and in Minnesota (USA). Since SMA is not associated with metabolic under or overproduction, the screening test relies on the demonstration of the homozygous absence of exon 7 in *SMN1*. The cost is limited to less than 2 euros/baby. The main issue remains the best strategy to treat positive case and how to manage patients with 4 copies. In this talk, we will give some insight about ethical, technical, economical and medical challenge related to this disease screening.

### I23. When Is a Treatment “Accepted”? Balancing Pros and Cons for Expensive and Invasive Treatment after Neonatal Screening

CornelMartinaAmsterdam University Medical Center, Clinical Genetics and Amsterdam Public Health Research Institute, 1007MB Amsterdam, The Netherlands

One of the traditional screening criteria is that there should be an accepted treatment. New therapies are emerging for e.g., spinal muscular atrophy (SMA). The pros and cons need to be balanced to answer the question whether these therapies are “accepted”. Arguments from the Netherlands policy debate on SMA treatment with Nusinersen will serve to identify ethical, legal, social and economic issues at stake. Nusinersen (Spinraza^®^) is an antisense oligonucleotide drug developed for the treatment of SMA for intrathecal administration. It was evaluated by the Dutch National Health Care Institute that advised that the price should drop by >85% to allow for reimbursement in the basic health care package. It is the first medication that has become available for this group of patients, therefore stakeholders must commit to making it available as soon as possible. Effectiveness was considered proven for patients with the most severe forms. Inferring evidence from one group to another was not considered valid. Long-term effects are still uncertain. The cost per Quality Adjusted Life Year (QALY) was estimated to be €600.000 for type 1 SMA and €1.7 million for type 2/3a SMA. For type 1 SMA the medication will never be cost-effective, even if provided for free, as a longer life will imply high medical cost (hospital admissions, ventilation). The producer has determined an extremely high price, without making transparent why this is reasonable and just. Producers should take their moral responsibility to make innovative medication available for all patients. Evidence, economics, and ethics all must be considered in developing screening policies. We need to attune responsibilities for public and private stakeholders.

### I24. Information for Parents and Professionals, Status in Europe

JansenMarleenFrankovaVeraKožichViktorLoeberGerardRIVM, 3720BA Bilthoven, The Netherlands

As neonatal screening innovates, countries increasingly follow in the footsteps of existing screening programs and develop a program suitable for their local context. Information is readily available on the evaluation of conditions appropriate for screening, both in academic literature and government websites. This information enables valuable learning from other neonatal screening programs on what conditions to screen for. However, when it comes to the practical implications of initiating a screening program or implementing a new condition to an existing program, the information becomes harder to gather. For example, adequate parental and professional information appears to be ‘reinventing the wheel’ for many local screening programs. The aim of this project is to develop a format for information for parents to aid countries that start a new program or implement an additional condition, potentially with limited access to international information. To facilitate a format for parent information, information products from different countries were collected. When needed, they were translated to English. The different products were compared on topics in the general information, the amount of detail in explaining included conditions, and the types of products, such as leaflets and animations. Each country or region was involved by contacting a contact person of the neonatal program through the project team’s network, to enable distributing a short survey on the development of the information product(s) and a member check of the analysis. The format includes main topics for general information, with examples from different countries. Suggestions for the amount of detail in the information per included condition, again based on examples from different countries. In line with the ISNS mission, the information will be disseminated through a format available from the ISNS-website.

### I25. Guidelines and Practice of Newborn Screening for Homocystinuria

R.Keller^1^,^2^P.Chrastina^3^M.Pavlíková^3^,^4^S.Gouveia^5^A.Ribes^6^S.Kölker^7^J.Blom H.^8^R.Baumgartner M.^1^,^2^J.Bártl^3^C.Dionisi Vici^9^F.Gleich^7^A.Morris A.^10^V.Kožich^3^M.Huemer^1^,^2^,^11^Individual Contributors of the European Network and Registry for Homocystinurias and Methylation Defects (E-HOD)1Division of Metabolism and Children’s Research Center, University Children’s Hospital Zürich, 8032 Zürich, Switzerland2Radiz–Rare Disease Initiative Zürich, clinical research priority program, University of Zürich, 8032 8032 Zürich, Switzerland3Department of Pediatrics and Adolescent Medicine, Charles University-First Faculty of Medicine and General University Hospital, 10000 Prague 10, Czech Republic4Department of Probability and Mathematical Statistics, Charles University-Faculty of Mathematics and Physics, 10000 Prague 10, Czech Republic5Unit of Diagnosis and Treatment of Congenital Metabolic Diseases, S. Neonatology, Department of Pediatrics, Hospital Clínico Universitario de Santiago de Compostela, CIBERER, Health Research Institute of Santiago de Compostela (IDIS), 15706 Santiago de Compostela, Spain6Division of Inborn Errors of Metabolism, Department of Biochemistry and Molecular Genetics. Hospital Clinic de Barcelona, CIBERER, 08306 Barcelona, Spain7Division of Neuropaediatrics and Metabolic Medicine, Center for Pediatric and Adolescent Medicine, University Hospital Heidelberg, Heidelberg 69120, Germany8Department of internal medicine, VU Medical Center, 1007 MB Amsterdam, The Netherlands9Division of Metabolism, Bambino Gesù Children’s Research Hospital, 00165 Rome, Italy10Manchester Center for Genomic Medicine, Manchester University Hospitals NHS Trust, Machester M13 9WL, UK11Dept. of Pediatrics, Landeskrankenhaus Bregenz, 6900 Bregenz, Austria

Several guidelines on screening for homocystinuria have been developed recently. In this study, we assessed how the current practice of newborn screening (NBS) for homocystinuria compares with published recommendations. Twenty-two of 32 NBS programs from 18 countries screened for at least one form of homocystinuria. Centers provided pseudonymized NBS data from patients with deficiency of cystathionine beta-synthase (CBSD, *n* = 19), methionine adenosyltransferase I/III (MATI/IIID, *n* = 28), combined remethylation defects (cRMD; *n* = 56) and isolated RMD (iRMD) including methylenetetrahydrofolate reductase deficiency (MTHFRD) (*n* = 8). Markers and decision limits were converted to multiples of the median (MoM) to allow comparison between centers. NBS programs, algorithms and decision limits varied considerably. Only nine centers used the recommended second-tier marker total homocysteine (tHcy). Median decision limits of all centers were ≥2.35 for high and ≤0.44 MoM for low methionine; ≥1.95 for high and ≤0.47 MoM for low methionine/phenylalanine, ≥2.54 for high propionylcarnitine and ≥2.78 MoM for propionylcarnitine/acetylcarnitine. These decision limits alone had a 100%, 100%, 86%, and 84% sensitivity for detection of CBSD, MATI/IIID, iRMD and cRMD, respectively but failed to detect six individuals with cRMD. To enhance sensitivity and decrease second-tier testing costs we further adapted these decision limits using data of 15,000 healthy newborns. Current practice of NBS for homocystinuria is lagging behind the recent recommendations. Due to the favorable outcome of early treated patients, NBS for homocystinuria is recommended. To improve NBS, decision limits should be revised (considering the population median) and relevant markers should be combined. Use of the post-analytical tools offered by CLIR (which considers e.g., birth weight and gestational age) and implementation of tHcy and MMA as second-tier markers should be considered.

## 3. Oral Communications

### O1. Newborn Screening for X-Linked Adrenoleukodystrophy, the Chop Experience

FiciciogluCanThe Children’s Hospital of Philadelphia, Perelman School of Medicine at the University of Pennsylvania, Pediatrics and Human genetics, Philadelphia, PA 19104, USA

X-linked adrenoleukodystrophy (X-ALD) is a metabolic disorder caused by mutations in ABCD1. In X-ALD, very long-chain fatty acids (VLCFAs) cannot be transported into peroxisomes to be broken down and accumulating VLCFAs damage the nervous system and the adrenal glands. X-ALD affects 1 in 17,000 individuals worldwide, regardless of race, ethnicity and geography. X-ALD has a wide range of disease severity, but as an X-linked condition, generally affects males more severely. Based on the availability of therapy, public health programs are beginning to implement newborn screening (NBS) using dried blood spot analysis for X-ALD. Screening females identifies older unscreened male siblings who may still benefit from intensive surveillance protocols if found to have X-ALD, as well as males in the extended pedigree at risk for adrenomyeloneuropathy and isolated adrenal insufficiency forms of the disease. Pennsylvania began NBS for X-ALD in April 2017. All infants born in Pennsylvania since the program start date were screened via high-throughput standard MS/MS analysis of C26:0 lysophosphatidylcholine (C26:0-LPC) on NBS dried blood spot specimens. Repeat samples were required for any neonate with elevated C26:0-LPC. If C26:0-LPC levels remained elevated, full ABCD1 gene sequencing was completed and patients were referred to a metabolic center for further evaluation. Newborn screening for X-ALD in Pennsylvania has already identified several patients during the first 12 months of testing including a case with peroxisomal biogenesis defect. We report here a retrospective analysis of the clinical and laboratory features of neonates with positive screen referred to our center.

### O2. Increased Use of Pivmecillinam Highlights the Need for a 2nd Tier Screening Test to Improve Analytical Reliability of Isovaleric Acidemia Screening

CarlingRachelBurdenDebbieJohnKateBonhamJimViapath, Guys & St Thomas’ NHSFT, Biochemical Sciences, London SE1 7EH, UK

England has been screening for Isovaleric Acidemia (IVA) by flow injection analysis tandem mass spectrometry of isovaleryl-carnitine since 2015. Isovaleryl-carnitine is isobaric with pivaloylcarnitine which can be present in blood due to pivalic ester pro-drugs such as Pivmecillinam, a broad-spectrum antibiotic used to treat urine infections, or pivalic acid derivatives found in some nipple balms. The potential for false positive (FP) results is well known. The cut-off value is 2 µmol/L, 4.5 times higher than that recommended by CLIR. There have been 19 condition-suspected results to date, 6 of which were true positives. We developed a method to measure pivaloylcarnitine in these samples. Dried bloodspots were eluted, and the extract injected into a Waters Acquity UPLC coupled to a Premier XE mass spectrometer. Isocratic separation of isovaleryl-carnitine, pivaloylcarnitine, valerylcarnitine and 2-methylbutyrylcarnitine was achieved. Intra and interassay precision were <6% and it was linear to 10 µmol/L. All 13FP were due to the presence of pivaloylcarnitine. 8/13 had a history of maternal antibiotics, 1/13 was using Mustela nipple balm and limited information was available on 4/13. The incidence of FP reflects the prescribing patterns of Pivmecillinam, use of which has recently increased due to antibiotic resistance. Guidance from Public Health England in 2016 recommends it as an alternative therapy if there is widespread bacterial resistance to ampicillin, amoxicillin and trimethoprim; Pivmecillinam prescriptions in England have risen sharply from 27,614 in 2012 to 483,790 in 2017. If this trend continues, so will the incidence of FP results. Introducing pivaloylcarnitine as a 2nd tier screening test will prevent unnecessary referrals and improve the reliability of IVA screening.

### O3. Sickle Screening by MSMS: The Practicalities of Introducing into the Routine Newborn Screening Service

JohnKateViapath, St Thomas’ Hospital, Biochemical Sciences, London SE1 7EH, UK

Screening for sickle cell anemia was first introduced in the UK in 1978 and rolled out nationally in 2006 with the majority using HPLC. By March 2018, 4 labs have moved to FIA-MSMS. We detail our experience validating the method on 2 Waters Xevo TQ-S analyzers and introducing the service into routine use. The validation was undertaken over an 18-month period during which time 7745 blood spots, including 518 with Hb variants, were analyzed and compared with results obtained by HPLC. All clinically relevant conditions were identified correctly; however, the validation was not without several significant issues. In the initial validation of 3012 samples 109 had elevated F:A ratios and 29 false positive HbD^Punjab^ were detected. Subsequently, when samples were analyzed in parallel on both instruments, the F:A ratio, HbC and E did not compare well which was problematic for values around the action limits; at one stage 58 false positives were identified for HbE on one of the instruments. Throughout the validation as batches were analyzed the sensitivity gradually deteriorated which affects each peptide differently thereby altering the ratios. To solve these issues various steps were taken. Probe position was altered; alternative daughter ion chosen for HbD; specifically, ‘tuned’ certain peptides to match between both analyzers; changed the ion source and cleaned the LC. These last two steps must be undertaken periodically to avoid a drop-in sensitivity, particularly important for labs who do not have dedicated screening instruments. It is evident that screening for Hb variants by MSMS is challenging. However, the technology can and does identify those HbS of interest and is undoubtedly going to be the method of choice in the future. Our lab went live in March 2018.

### O4. Simultaneous Transition to a New Tandem Mass Spectrometry System in Five (Dutch) Laboratories: Experimental Evaluation to Monitor Analytical Changes

BouvaMarelleMaaseRoseVeen-SijneMarja vanBoelenAnitaRijswijkCarolien vanKemperEvelienBurgmansLenyJakobsBernadetteBeltmanGerdaEngelHenkWeijmanGertSchielenPeterNational Institute for Public Health and the Environment, Center for Health Protection, NL-3720 BA Bilthoven, The Netherlands

All five Dutch Newborn Screening laboratories changed from a Waters Quattro Micro with Perkin-Elmer NeoBase combination to a Waters Xevo TQD with Perkin-Elmer Neobase2 (Xevo) combination on 2 January 2018. The validation study in 2017 showed a shift in measured concentrations for some parameters: for succinylacetone (SA), the cut-off value was changed from 1.2 to 0.9 µmol/L blood to reflect the reduced measured concentration. A protocol was initiated in all five screening laboratories to monitor measurement levels and performance of the Xevo prior to January 2018. Thirteen plates (approx. 1200 samples) were analyzed over a period of seven weeks. Samples were routine screening samples, NeoBase and Neobase2 kit controls, CDC proficiency testing samples, previous screen positive samples and so-called multi-level control samples provided by the kit-manufacturer. The results of the analyses showed continuity of performance, but compared to the validation study, a more marked decrease in the measured concentrations of some parameters. For these parameters, samples with a concentration near the cut-off values (in a defined ‘grey area’) were evaluated with extra scrutiny, to decrease the risk for false negative screening results. In January and February 2018, there were 26 referrals for conditions detected using the Xevo. Seven of these were based on screening results in the grey area and of these, six were confirmed false positive. Frequency distribution of measured concentration showed, for all parameters except SA, a narrower, but slightly shifted distribution compared with the first three months of 2017. Despite extensive evaluation of a new method, unexpected shifts in measured concentrations can appear. Continued regular evaluation of grey area results and referrals is in place to mitigate false negative or false positive results.

### O5. Improving Dried Blood Spot Quality in Preparation of the Expansion of the Neonatal Screening Program in the Netherlands

Verschoof-PuiteRendelienWeijmansGertJansenMandyHeijnenMarie-LouiseBouvaMarelleRIVM, Department for Vaccine Supply and Prevention Programs, 3721 MA Bilthoven, The Netherlands

In December 2017 the Dutch State Secretary for Health decided to gradually expand the screening program in the Netherlands from 19 to 31 diseases. One of the challenges in the expansion is the amount of blood needed. The new analyses are preferably done on the current six dried bloodspots (DBS). Currently 1% of the DBS cards has insufficient blood for analysis. For these children a new heel prick is needed. An inquiry at the five screening laboratories showed that about 40% of the DBS cards will have insufficient blood to perform analyses for all 31 diseases. This shows the need to improve the quality of the bloodspots. Multiple initiatives were started to improve the quality of the DBS. To improve the blood withdrawals feedback was given to screeners, an e-learning for screeners was developed and face-to-face education was intensified. A European tender for a new lancet is carried out. Unique part of this tender was a field test: 18 screeners tested three lancets selected in the first part of the tender process. The DBS that resulted from these blood draws were judged on quality. One of the criteria was ‘sufficient blood to obtain 30 1/8-inch punches’. The quality of the DBS obtained with the three lancets, together with the opinion and experience of the screeners will determine the selection of a lancet. To measure the effect of actions taken, the screening laboratories will perform a new inquiry to evaluate the quality of bloodspots once the selected lancet is in use for a while. These results will be presented too.

### O6. Using a Common Internal Standard to Reduce Interlaboratory Variation and Improve Analytical Reliability of Expanded Newborn Screening in England

CarlingaRachelJohnKateViapath, Guys & St Thomas’ NHSFT, Biochemical Sciences, London SE1 7EH, UK

Since 2015, England has been screening for 6 disorders (PKU, MCADD, IVA, HCU, GA1, MSUD) by tandem mass spectrometry. The screening program is tightly governed: all 13 laboratories adhere to national protocols with common cut-off values (COV). It is important that interlaboratory variation is minimal to ensure analytical reliability of results. However, the lack of traceable reference materials, predominant use of in-house reagents and limitations of the existing methodology make this challenging. Interlaboratory variation at the 90th centile ranges from 17% for octanoylcarnitine to 50% for decanoylcarnitine. The situation is compounded by the use of ‘calibration factors’ in most laboratories. Factors range from 0.74 to 1.64. The evidence base for these factors is lacking and makes it difficult to separate out true differences due to instrument and reagent variation, from gross error. For most analytes there is good discrimination between the COV and the affected population so while there is minimal risk of false negative results due to inaccuracy, this is not necessarily the case for false positive results. We believe the key contributors to interlaboratory variation are instrument set up and internal standard. To investigate the latter, we distributed a common internal standard mix to 4 laboratories and screened 232,000 babies. The interlaboratory variation for all analytes was compared with that of 9 laboratories using in-house (*n* = 6) and commercial (*n* = 3) internal standard to screen 486,000 babies. Results indicated a common internal standard significantly reduced interlaboratory variation for most analytes. For example, the CV at the 90th centile for isovaleryl-carnitine common IS group was 8% vs. 24% for the in-house group, likewise glutarylcarnitine at 6% vs 23%.

### O7. Reliability of Newborn Screening in Preterm, Low Birthweight and Sick Infants

D.WebsterM.de HoraN.HeatherJ.and LuedkeNational Testing Centre, Auckland AK1, New Zealand

There are special challenges in obtaining reliable newborn screening results in a population of newborns quite unlike healthy term newborns, for example, reliable results for fatty acid oxidation and amino acid breakdown disorders rely on samples being taken in, or shortly after, the period of catabolism newborns have shortly after birth; an early aim for babies admitted to special and neonatal intensive care units is the prevention of catabolism. The hypothalamic-pituitary axis may not be fully developed at the time of birth so despite low thyroxine levels TSH may not be elevated in babies with congenital hypothyroidism leading to false negative screen results; the immune system may not be fully developed leading to false positive SCID screen results. Many programs schedule further samples on these low, and very low, birthweight infants and there is significant variation between program recommendations. The Clinical and Laboratory Standards Institute (CLSI) has produced a guideline to ensure rapid, consistent, and complete newborn screening to facilitate early diagnosis and treatment for the newborns affected with a screened condition and minimize the risk of a missed or delayed diagnosis; to optimize the timing and minimize the number of screening events and to define essential elements of quality assurance relevant to this population; provide education on the effects of special and intensive care unit treatments on newborn screening and identify areas needing further research. The revised guideline is due for publication and recommendations will be presented.

### O8. Severe Combined Immunodeficiency–Single Center Experience

ČižnárPeterHorákováJuliaBodováIvanaSvecPeterUrdováVeronikaNational Institute of Child Diseases, Pediatric Department, 83348 Bratislava 37, Slovak Republic

Severe combined immunodeficiencies (SCID) comprises a heterogeneous group of diseases characterized by severe defects in the development and function of T, B and NK lymphocytes. They are considered a pediatric emergency, as they lead to severe, life threatening infections in the first two years of life. Majority of children if treated adequately could be cured and have good quality of life. In the past 15 years 18 SCID cases have been documented in Primary Immunodeficiency Center at Children University Hospital in Bratislava. Final diagnosis was confirmed for X-SCID (common gamma chain deficiency) in 6 cases, adenosine deaminase deficiency in 4 cases, RAG deficiency in 3 cases, complete 22q11del syndrome in 1 case. In rest of the mostly earlier patients the genetic diagnosis was not established. In 11 cases allogenic hematopoietic stem cell transplantation was carried out (2 HLA identical, 2 matched unrelated donors), with survival rate 91%. The cause of death were severe infectious complications. Quantification technique TREC and qRT-PCR method have been already available for newborn screening. We have retrospectively analyzed all our SCID patient for the neonatal dry blood spots TRECs and confirmed low number of copies, pointing to a positive result if provided as a screening method. Eight out of 18 patients would have a better chance for survival if the newborn screening was applied in Slovakia.

### O9. Second-Tier Next Generation Sequencing in Newborn SCID Screening Provides Rapid Molecular Confirmation of Disease with Implications for Follow-Up and Treatment

Stray-PedersenAsbjørgStrandJanneLundmanEmmaGulKiran AftabAbrahamsenTore G.ØverlandTorsteinErichsenHans ChristianTangeraasTrineJørgensenJensKristiansenErleBergeMona K.MørkridLarsRootweltTerjeRoweAlexander D.PettersenRolf D.Oslo University Hospital, Norwegian National Unit for Newborn Screening, Oslo 0424, Norway

Severe combined immunodeficiency (SCID) and other T-cell deficiencies are identified by newborn screening (NBS) measuring T-cell excision circles (TRECs) by qPCR in dry blood spot DNA. Second-tier next generation sequencing (NGS) on the same dry blood spot DNA was introduced as part of our prospective pilot research project in 2015. Based on written informed consent, parents of newborns at selected hospitals were offered SCID screening. Among the 20,000 TREC-tested newborns, 3 individuals with SCID were identified. In addition, their disease-causing variants in IL2RG, RAG2, and RMRP, were found within 2 days after DNA extraction, utilizing rapid NGS with Ion PGMTM PID gene panel. The molecular findings directed follow-up and therapy: the IL2RG-SCID underwent early hematopoietic stem cell transplantation, without any complications; the RAG2-SCID received prophylactic antibiotics, antifungal treatment, and immunoglobulin infusions, and was successfully transplanted at 1 year of age. While the RMRP-individual died within 2 weeks of complete Hirschsprung. Twelve other newborns had TRECs below 20/μL but no pathological findings on gene panel testing, and other causes than primary immunodeficiency likely responsible for the low TRECs: 3 were born prematurely, 3 had congenital heart defects, 4 intestinal malformations, and 2 transient low TRECs and a history of failure to thrive after birth. In our retrospective study using neonatal blood spots from 18 patients with known immunodeficiencies, we were able to detect low TRECs and confirm the molecular diagnoses by NGS. In conclusion, TRECs as 1st tier, and NGS gene panel as 2nd tier integrated in the screening algorithm, promise rapid detection of newborns with SCID and other severe T-cell deficiencies. Our 2-step SCID-screening test strategy may reduce mortality and morbidity, minimize the time to definitive molecular diagnosis, reduce number of recalls and blood redraws, reduce false positives, and increase the positive predictive value. Since 1 January 2018 all newborns in Norway are offered SCID screening as part of the ordinary NBS program.

### O10. Probabilistic Uncertainty Analysis of Economic Models to Assess the Reliability of Screening Policy Decisions

ChilcottJamesBesseyAliceLeavissJoannaWongaRuthCruzCarmen de laUniversity of Sheffield, School of Health and Related Research, Sheffield S10 2TH, UK

To predict the economic impact of screening for severe combined immunodeficiency (SCID) in the UK NHS Newborn Blood Spot Screening Program. A decision analytic model with a public service perspective was used to estimate the number of SCID and non-SCID cases identified, lifetime costs and quality adjusted life years (QALYs). Systematic reviews and expert clinical judgment were used with probabilistic sensitivity analyses and threshold analyses to assess uncertainty in predictions. A threshold disbenefit analysis was used to assess the potential impact of false positives and healthy at birth non-SCID T-cell lymphopenia (TCL) diagnoses. It is estimated that screening for SCID in the UK’s 760,000 annual births would result in 310 (72–811) presumptive positive cases, 260 (25–760) false positives, 26 (9–50) non-SCID TCL cases and 17 (14–22) SCID cases. Screening was predicted to reduce mortality from 8 (5.3–12) to 1.7 (0.6–4.1) deaths and increase QALYs and costs with an incremental cost-effectiveness ratio of £17,642 per QALY gained. It was estimated that to push the cost-effectiveness over the £20,000 threshold the 6.5 (1.5–16) healthy at birth cases would need a disbenefit of 2 QALYs and the false positive cases a disbenefit of over 12 quality adjusted days. Screening for SCID is potentially cost-effective at a threshold of £20,000 per QALY, key uncertainties relate to the cost of the screening test, the incidence of SCID and the impact of false positives and the identification of children with non-SCID TCL.

### O11. Screening for Spinal Muscular Atrophy in North Carolina State

TaylorJenniferLeeStaceyShoneScottWheelerAnneOkoniewskiCaseyPowellCynthiaClinardKristinFanZhengBaileyDonBeckloffSaraRTI International, Center for Newborn Screening, Ethics, and Disability Studies, Research Triangle Park, NC 27709, USA

The National Institute for Child Health and Human Development awarded a contract to RTI International, the University of North Carolina at Chapel Hill, and the North Carolina State Laboratory of Public Health to conduct a newborn screening pilot study for spinal muscular atrophy (SMA). SMA type I is consider the most common lethal autosomal recessive genetic disorder in infants and SMA was recently recommended to the Recommended Uniform Screening Panel (RUSP) in February 2018. We validated a real-time qPCR assay to detect the presence or absence of the SMN1 exon 7 deletion, a method adapted from the previous Taylor et al. publication. We characterized the performance of this method by evaluating assay precision, accuracy, sensitivity, and specificity. The validation study included positive controls with known SMN1/SMN2 genotypes and 2000 de-identified dried blood spots (DBS). SMA screening will be included in a voluntary screening program called Early Check in the summer of 2018. Early Check offers screening to all families state-wide in North Carolina for a panel of conditions. This presentation will show the SMA validation results, the implementation experience, as well as screening results. The results from this study support the application of this method for high-throughput newborn screening programs and screening for SMA facilities the early identification of infants that will receive clinical services.

### O12. Validation of a Fast, Robust, High-Throughput, Inexpensive, First-Tier Molecular Neonatal Screening Test on Dried Blood Spots for Spinal Muscular Atrophy without Carrier Detection

StrunkAnnuska^1^AbbesAndre^1^StuitjeToon^2^HettingaChris^2^SepersEline^2^SnetselaarReinier^2^SchoutenJan^2^AsselmanFay-Lynn^3^CuppenInge^3^LemminkHenny^4^PolLudo van der^3^EngelHenk^1^1Department of Clinical Chemistry and Neonatal Screening, Isala Hospital, 8025 Zwolle, The Netherlands2MRC-Holland, 1057 DL Amsterdam, The Netherlands3Department of Neurology, Brain Center Rudolf Magnus, University Medical Center Utrecht, 3584 CX Utrecht, The Netherlands4Department of Genetics, University Medical Center Groningen, 9713 GZ Groningen, The Netherlands

Spinal Muscular Atrophy (SMA), in >95% of SMA cases caused by the homozygous deletion of the survival motor neuron (SMN1) 1 gene, is with an incidence of 1:10,000 one of the leading genetic causes of infantile mortality. Recently introduced antisense oligonucleotide treatment (AON) improves outcome, but has a narrow window of opportunity. Presymptomatic disease detection is therefore vital. We developed and validated a new SALSA^®^ MC002 melting curve assay that meets Dutch legal requirements of not detecting asymptomatic carriers. It detects the absence of the SMN1 exon 7 DNA sequence using crude extracts from dried blood spot (DBS) newborn screening cards. Melting curve analysis shows specific peaks of both the SMN1 and disease modifying SMN2 homolog or, in case of insufficient amounts of sample DNA, a warning peak. We retrieved 47 DBS samples from children with genetically confirmed SMA (identified by using the Dutch SMA database) after consent from parents and 375 controls from the national archive of the Dutch National Institute for Public Health and the Environment (RIVM). The assay correctly identified all anonymized SMA and control samples (i.e., sensitivity and specificity of 100%), without the detection of carriers, on 3 most commonly used PCR platforms with melting curve analysis. Concordance with the second-tier “golden standard” SALSA^®^ MLPA P060-B2 and P021-X1 test was 100%. SMN2 copy number determination from DBS with the MLPA P021-X1 test shows a concordance of at least 93%. The MC002 test showed feasibility and accuracy for SMA screening in a neonatal screening program and can be performed in crude extracts from a single 1.5 or 3.2 mm punch.

### O13. (S)un (M)ay (A)rise on SMA: The Newborn Screening Liege’s Experience

BoemerFrançois^1^CabergJean-Hubert^2^DangouloffTamara^3^,^4^ServaisLaurent^3^,^4^1Biochemical Genetics Lab, Department of Human Genetics, CHU of Liege, University of Liege, Liege 4000, Belgium2Molecular Genetics Lab, Department of Human Genetics, CHU of Liege, University of Liege, Liege 4000, Belgium3I-Motion, Platform for Pediatric Clinical Trials, Arnold Trousseau Hospital, 75012 Paris, France4Neuromuscular Center, CHR Citadelle, University of Liege, Liege 4000, Belgium

Considering the effectiveness of new therapeutic approaches for Spinal Muscular Atrophy when administered early and the societal burden of Spinal Muscular Atrophy-related disability, the implementation of a newborn screening program for Spinal Muscular Atrophy (SMA) currently appears as ethical and medical evidence. We have thus developed a newborn screening method to specifically recognize affected-SMA neonates. Our analytical methodology relies on a qPCR assay of the *SMN1* gene on DNA extracted from DBS, using *RPP30* as the reference gene. *SMN1* genotyping was designed to detect only homozygous deletions of exon 7 with a specific locked nucleic acid (LNA) probe. Our assay does not identify heterozygous carriers of the deletion, *SMN1* point mutations or the number of copies of the *SMN2* modifier gene. The assay was subsequently validated against 53 homozygous patients and 93 heterozygous relatives sampled on dried blood spots. To interpret the results on a larger scale, the *SMN1* results were integrated with the *RPP30* amplification results by calculating endpoint-fluorescence ratios. We have now initiated a 3-year pilot study (ethic approval: B412201734396) in the Liege’s newborn screening laboratory to initially cover 17,000 neonates per year (coverage extension to all of southern Belgium is underway). To date, about 7200 neonates have been screened. Two positive cases were identified and confirmed using Multiplex Ligation-dependent Probe Amplification (MLPA) technique. Patients were referred for pre-symptomatic care to our center of neuromuscular disorders. Expenditures dedicated to implanting our SMA screening assay are very reasonable and do not exceed the costs of other commonly accepted screenings.

### O14. Factors Influencing Parental Awareness about Newborn Screening

FrankovaVeraKutarnaAndrejHermankovaRenataJešinaPavelDohnalovaAlenaPeškováKarolínaKožichViktorCharles University-First Faculty of Medicine, Dept. of Pediatrics and Adolescent Medicine, Praha 2, Czech Republic

Appropriate and timely education about newborn screening (NBS) contributes to parental satisfaction with, and trust in the NBS. It also helps to foster the benefits (e.g., prompt follow-up), promote parents’ autonomy via informed consent and minimize the harms such as reducing the impact of NBS false positive results. The aim of this study was to ascertain how mothers are informed about the NBS in the Czech Republic and to identify variables associated with the awareness about NBS. A random sample of 3000 mothers was invited to participate in the study. The questionnaires evaluating the awareness and its determinants were mailed to mothers 3 months after the delivery. The overall response rate was 42%. We analyzed 1100 questionnaires and we observed that better awareness about NBS has been significantly associated with age, parity, number of information sources, child health status, size of maternity hospital and obstetrician as the source of information (=information received prenatally). Although majority of mothers (77%) in our study recalled being informed by a physician or nurse in neonatal ward, results have revealed that over 40% of participants did not have sufficient awareness about the principal aspects of NBS. Several measures including seminars for healthcare providers, development and distribution of new educational materials were adopted to improve the parental education on NBS in the Czech Republic. This study was supported by project RVO VFN 64165

### O15. “So Your Baby Had an Abnormal Neonatal Screen Result. Now What?”: Keys to Effective Communication

ViallSarahChildren’s National Health System, Genetics and Metabolism, Washington, DC 20010, USA

As neonatal screening programs become more widespread, thousands of newborns will receive diagnoses of life-long medical conditions which will benefit from rapid treatment. In addition, as an incontrovertible result of population-based screening implementation, many children will have false positive screens which can lead to significant family anxiety and frustration with the program. Screening stakeholders must understand the integral role of appropriate communication when connecting these families with timely, expedient diagnostic testing and follow-up. In addition, participants across all settings, including laboratory staff, public health staff, primary and sub-specialty healthcare providers must be knowledgeable and comfortable discussing the clinical and ethical implications of abnormal neonatal screen results for families and their newborns.

References

1. Buchbinder, M.; Timmermans, S. Newborn screening for metabolic disorders: Parental perceptions of the initial communication of results. *Clin. Pediatr.*
**2012**, *51*, 739–744, doi:10.1177/0009922812446011.
2. Schmidt, J.L.; Castellanos-Brown, K.; Childress, S.; Bonhomme, N.; Oktay, J.S.; Terry, S.F.; Kyler, P.; Davidoff, A.; Greene, C. The impact of false-positive newborn screening results on families: A qualitative study. *Genet. Med.*
**2012**, *14*, 76–80, doi:10.1038/gim.2011.5.
3. Tluczek, A.; De Luca, M. Newborn screening policy and practice issues for nurses. *J. Obstet. Gynecol. Neonatal Nurs.*
**2013**, *42*, 718–729, doi:10.1111/1552-6909.12252.

### O16. Early Check: A Voluntary Public Health Research Program to Inform Newborn Screening

ShoneScottBieseckerBarbaraBoyeaBethDuparcMartinEdwardsAnneGehtlandLisaHarperBlakeLeeStaceyOkeniewskiCaseyPeayHollyRaspaMelissaTaylorJenniferWheelerAnneBaileyDonaldRTI International, Newborn Screening, Ethics, and Disability Studies, Research Triangle Park, NC 27709, USA

Newborn screening (NBS) is designed for pre-symptomatic identification of newborns with conditions for which there are effective treatments that must begin early. Central to NBS policy is reliable evidence that pre-symptomatic treatment is more effective than treatment after symptoms appear. This evidence is difficult to amass because most conditions are rare and not diagnosed early. Thus, researchers and advocates find themselves in a classic “Catch 22” situation—NBS for a condition will not happen without sufficient evidence, but gathering this evidence requires extensive population screening. Early Check (EC) is a large-scale, voluntary research program that screens newborns in North Carolina (NC) for conditions that are not yet nationally recommended to study the benefits of early identification and pre-symptomatic treatment. Recruitment will begin in spring 2018 to screen for 2 conditions: fragile X syndrome and spinal muscular atrophy. Mothers of the more than 120,000 babies born in NC each year can give permission while pregnant or before the newborn is 4 weeks old using an electronic consent module. Screening is performed on residual dried blood spots from NC’s NBS laboratory, and all newborns who screen positive for a condition on the EC panel will receive diagnostic testing. Those newborns who are confirmed with a disorder will be provided with genetic counseling, baseline medical and developmental assessments, and access to available treatment options. Families will also be asked to join the EC Research Registry, which will be used for natural history studies and to facilitate access to future clinical trials or other treatment options. Data will be presented from the first few months of EC, including recruitment, screening results, and diagnostic testing.

### O17. Lysosomal Storage Disorder Newborn Screening: Comparing Performance of Tandem Mass Spectrometry and Digital Microfluidics Platforms

MillingtonDavidBrennanCandiceKanungoManasPamulaVamseeDuke University Health System, Pediatrics, Durham, NC 27713, USA

Newborn screening (NBS) for lysosomal storage disorders (LSDs) has gradually gained momentum over the past decade and live screening is already active in a handful of US states for Pompe disease and Mucopolysaccharidosis Type I (MPS-I). Two platforms are currently available for high-throughput LSD enzyme testing from dried blood spot specimens–tandem mass spectrometry (MS/MS) and digital microfluidic fluorometry (DMF), which is authorized by the US FDA and CE Marked for LSD testing in other countries. Both platforms offer specific reagent kits, use synthetic substrates, and can screen for multiple LSDs in a single run. Regarding the analytical range of each platform, we caution that this metric has limited relevance to the ability of each method to discriminate normal from affected samples because it does not consider the multiple sources of variability (biological variability, DBS sample quality, gestational age at sampling, etc.) that exist between newborn samples. Indeed, rich output data sets from the prospective LSD screening programs in Missouri (DMF) and Illinois (MS/MS) are now available, and do not show significant differences between either platform in terms of the positive predictive value. We conclude that the active and pilot LSD screening programs in the United States are excellent, unbiased resources for those interested in adding newborn screening for LSDs. The emerging clinical results from these prospective screening programs reflect the real-world performance of each platform and should be considered alongside the associated costs (equipment, maintenance, personnel, etc.) and workflow of each platform to determine the best fit for each NBS laboratory.

### O18. Quality Improvement Facilitation via NBS Program Site Visits Offered by APHL

ZarbalianGuisouAssociation of Public Health Laboratories, Newborn Screening and Genetics, Silver Spring, MD 20910, USA

The Association of Public Health Laboratories (APHL) Newborn Screening and Genetics (NBSG) Program facilitates quality improvement initiatives across US newborn screening (NBS) programs by conducting site visits and other technical assistance activities. Objectives: To describe common themes and specific improvements that states have been able to implement because of such visits. Two types of site visits are offered, both relying on collaboration with subject matter experts (SME) and federal partners to be successful and productive. Molecular Assessment Program (MAP) visits assess the laboratory’s molecular testing capabilities. Laboratory workspace, staffing needs, standard operating procedures (SOPs), and other relevant documents and factors are evaluated to suggest areas for improvement. Representatives from the Centers for Disease Control and Prevention’s (CDC) Molecular Quality Improvement Program (MQIP), SME from other state NBS programs, and APHL NBSG program staff compose the assessment team. SME are chosen based on their specific expertise and the needs specified by the requesting laboratory. The Newborn Screening Technical Assistance and Evaluation Program (NewSTEPs) also conducts site visits, similar to the MAP visit model but include the entire NBS laboratory, follow-up program, and may also involve birthing centers within the state. Suggested improvements are provided via a comprehensive report to the laboratory NBS program which may be used to justify recruitment, purchases, or other decisions requiring administrative approval. NBS programs have implemented many quality improvement measures (e.g., laboratory space optimization, timeliness improvements) using said reports. The MAP and NewSTEPs site visits exemplify one of many ways APHL collaborates with federal partners and SME from other state NBS programs to strengthen the role of NBS laboratories.

### O19. Smartphone Point-Of-Care Newborn Screening for Endocrine and Hematologic Disorders: Indications, Regulations, Experience, and Future Directions

EhrenkranzJoelMusinguziMosesWiessnerPollyUniversity of Colorado School of Medicine, Division of Endocrinology, Salt Lake City, UT 84107, USA

Newborn screening represents a major public health achievement, as early diagnosis leads to prompt treatment, which, in turn, decreases morbidity and mortality. However, current newborn screening programs rely on centralized laboratory screening that requires infrastructure for specimen transport, laboratory analysis, results reporting, and follow-up. As a result, regions of the world that lack this infrastructure, including the Global South, Indian subcontinent, and remote and resource-limited areas, are unable to provide newborn screening services. Recently developed smartphone point-of-care (POC) immunochromatographic assays for TSH, cortisol, and sickle cell hemoglobinopathies provide an alternative method to central lab testing for newborn screening. POC newborn screening eliminates the need to transport large number of samples to a central laboratory, decreases the requirement for capital-intensive laboratory equipment, and shortens turn-around-time for results reporting and patient follow-up. Using a smartphone for patient registration, test interpretation, results reporting, and data archiving enables real-time geomapping, patient-specific decision support, and automated case management. We have used smartphone POC TSH testing for newborn thyroid screening in a Bangalore, India slum and in western Uganda, POC cortisol testing to screen for Addison’s disease in Kalahari Desert Bushmen, and POC sickle cell screening in a rural population on the Uganda Congo border. These studies demonstrate the feasibility and cost-effectiveness of POC newborn screening for endocrine and hematologic diseases, define the advantages, benefits, and limitations of POC newborn screening, and provide a template for developing POC methods to screen for other common congenital metabolic disorders such as PKU.

## 4. Posters

### P01. Evaluation of Selective Newborn Screening for Inborn Errors of Metabolism in Macedonia during the Period 2014–2017

AnastasovskaVioletaKocovaMirjanaPediatric Clinic, Laboratory for neonatal screening, Department of Endocrinology and Genetics, Skopje 1000, Macedonia

The introduction of tandem mass spectrometry (LC/MS/MS) in newborn screening programs in many countries, has increased the capacity to test newborns for inborn errors of metabolism (IEM). LC/MS/MS can identify and quantify several acylcarnitines and amino acids in a single test, and is able to detect more than 40 metabolic disorders, with the combined incidence of about 1 in 5000 babies, not including phenylketonuria (PKU). Screening for IEM in Macedonia was performed by measuring of two groups of compounds, 12 amino acids and 13 acylcarnitines (Chromsystems Diagnostics, Germany), in dried blood spot (*n* = 16,075) collected 48 h after births, using LC/MS/MS method, during the 2014–2017. Total of 16,075 newborns (18.6% of neonatal population per year), selected from six birth centers in the country (coverage 89.2%), have been screened. 8407 (52.3%) were male and 7668 were (47.7%) female with male to female ratio of 1.09:1. Among screened neonates 56.2% were Macedonians of Slavic origin, followed by ethnic Albanians (31.8%), Roma (6.7%), Turks (3.3%), Bosnians (0.7), Boshnjac (0.6%) and others (0.7%). Were detected total of 8 newborns with IEM: 4 with medium-chain acyl-CoA dehydrogenase deficiency (MCAD), 2 with phenylketonuria (PKU), one with hypermethioninemia (MET), and one with tyrosinemia type I (confirmed by the second-tier test). Selective screening is an important diagnostic tool for the diagnosis of various types of IEM, and it can provide substantial benefits to patients and their families. Early diagnosis is important not only for treatment but also for genetic counseling. Activities to cover all newborns in Macedonia are underway.

**Keywords:** Metabolic disorders; Newborn screening; Tandem mass spectrometry

### P02. Newborn Screening in the Czech Republic: Results from the Last 8 Years

VotavaFelixChrastinaPetrKožichViktorPeškováKarolínaAdamTomášFriedeckýDavidHlídkováEvaVinohradskáHanaHedelováMonikaDavidJanHolubováAndreaJr.Milan MacekSkalickáVeronikaGaillyováRenataValáškováIveta3rd Faculty of Medicine, Charles University and University Hospital Kralovske Vinohrady Prague, Pediatrics, Prague 10, Czech Republic

The nationwide newborn screening (NS) in the Czech Republic covers the whole newborn population and aids at detecting congenital hypothyroidism (CH), congenital adrenal hyperplasia (CAH), cystic fibrosis (CF), phenylketonuria/hyperphenylalaninemia (PKU/HPA), leucinosis (MSUD), glutaric aciduria type I (GA I), isovaleric aciduria (IVA), medium-chain acyl-CoA, long-chain 3-hydroxyacyl-CoA and very long-chain acyl-CoA dehydrogenase deficiency (MCADD, LCHADD and VLCADD), carnitine palmitoyltransferase I and II deficiency (CPTD I and II) and carnitine-acylcarnitine translocase deficiency (CACTD); since VI/2016 also argininemia (ARG), citrulinemia (CIT), biotinidase deficiency (BTD) and homocystinuria (CBS, MTHFR). Here we report data from the period I/2010–XII/2017. A total of 888,891 newborns were screened by use of fluoroimmunoassay for thyreotropin, 17-hydroxyprogesterone and immunoreactive trypsinogen (IRT). The CFTR gene (32 and later 50 mutations) was analyzed in 8859 (0.997%) blood spots with the highest IRT levels. Amino acids and acylcarnitines were analyzed by tandem mass spectrometry, biotinidase activity was determined in 181,396 newborns by fluorimetric assay. We detected 770 patients (cumulative detection rate 1:1034). The screening prevalence for individual disorders was as follows: CH 1:2914; CAH 1:12,520; CF 1:6683 (CF-SPID 1:22,792); PKU/HPA 1:5521; MCADD 1:22,792; LCHADD 1:80,808; VLCADD1:222,223; GA I and IVA 1:177,778; MSUD 1:296,297; CIT and CBS 1:181,396; and BTD (partial) 1:8638. No cases of other disorders were detected. NS is an effective approach for pre-symptomatic detection of serious rare diseases. Further optimization is needed. Supported by: PROGRES Q36 for FV.

### P03. Newborn Screening–Current Status in Alberta, Canada

BattochioAngieYuen-JungJoleneLilleyMargaretBlumenscheinPamelaRidsdaleRossSosovaIvetaUniversity of Alberta Hospital, Alberta Health Services, Newborn Metabolic Screening and Biochemical Genetics Laboratory, Edmonton, AB T6G 2B7, Canada

In Alberta, newborns are screened for 17 disorders (14 metabolic conditions, 2 endocrine conditions and cystic fibrosis). As of 1 April 2019, 4 additional disorders (galactosemia, tyrosinemia type 1, severe combined immunodeficiency and sickle cell disease) will be added to the newborn screening (NBS) panel. The NBS program is voluntary and parental refusal is permitted. The provincial health ministry (Alberta Health) sets the policies and standards for the program which is administered through Alberta Health Services, a province-wide integrated health system. A comprehensive Clinical Policy suite of documents sets out responsibilities and expectations for program delivery. All infants born in Alberta are registered and given an Alberta Unique Lifetime Identifier (ULI) within 24 h of age. NBS samples are collected between 24 h and 3 days (although the upper screening age limit is 2 years). A repeat screen between 21 and 28 days of age is required for newborns.

### P04. Past, Present and Future Perspectives of the Hellenic Newborn Screening Program

PlatisDimitrisGirginoudisPanagiotisSchulpisKleopatraKanaka-GantenbeinChristinaDoudounakisStavrosInstitute of Child Health, Biochemistry, Athens 11527, Greece

The Hellenic Newborn Screening program is solely implemented by the Institute of Child Health (ICH), a multidisciplinary institution supervised and funded by the Ministry of Health and Welfare. The program was initiated in 1974 for Phenylketonuria (PKU) covering the Athens Metropolitan area but quickly expanded to full national coverage (over 90,000 neonates/annum) and three additional conditions (Galactosemia, congenital hypothyroidism (CH) and Deficiency of G6PD). Over the last 40 years, more than 3,700,000 neonates have been screened. In a plethora of cases, ICH is not only responsible for final diagnosis and treatment initiation but also clinical follow-up of patients. In 2017, a new era was ushered with the creation of a unified Newborn Screening Department responsible for the laboratory testing of neonatal sample. This was accompanied by the arrival of a fully automated, high-throughput instrument (GSP, Perkin-Elmer) for the simultaneous screening of PKU, Galactosemia and CH, a donation by Stavros Niarchos Foundation, along with a needed upgrade to the Laboratory Information System (LIS), for automatic transfer of our data in a faster, safer and more accurate manner. The previously mentioned upgrades lead the way towards the introduction of cystic fibrosis in our screening roster, scheduled to begin by 2020. Despite our technical upgrades, a statistical analysis in 2015 of neonatal screening data (*n* = 92,657) revealed significant problems regarding harmonization of newborn screening protocols (e.g., Guthrie card expiration, failure to collect blood spots or to post cards on time, etc.) which can impact results significantly. Greater visibility of our work as well as implementation of informative campaigns are necessary to improve newborn screening framework compliance.

### P05. Screening for Inherited Metabolic Diseases in Moscow

PechatnikovaNataliaShestopalovaElenaPotehinOlegDenisenkovAlexanderBullikhArtemKakaulinaVictoriaPolyakovaNinaBruhanovaNataliaBaydakovaGalinaKoltunovIgorGbih Morozov’s Children Clinical Hospital, Inborn Errors of Metabolism, Moscow 125032, Russia

In Russia since 2006 all newborns are screened for 5 diseases: PKU, congenital hypothyroidism, galactosemia, congenital adrenal hyperplasia, cystic fibrosis. In 2016 high-risk screening to identify children with inherited metabolic diseases (IMD) was started in Moscow. In 2018 the newborn screening was extended for followed diseases: GA I, MSUD, MMA/PA, TYR-I, MCADD, BTD. Newborn screening was performed by immunofluorescence analysis (AutoDelfia1235 with DELFIA Express kit (Perkin-Elmer, Turku 20101, Finland)) and MS/MS on Waters TQD (Waters, Milford, MA 01757, USA) using NeoBase Mass Spectrometry Kit (Perkin-Elmer, Finland) with MassLynx software. The high-risk screening for IMD in children (0–18 years old) was performed by the MS/MS. Indications for the screening were specific clinical symptoms. Confirmatory tests included organic acids analysis, enzymes assays, and molecular genetic testing. From 2006 to 2017 in total 1,566,316 newborns were screened. The average frequency of screened IMD in Moscow is: PKU 1:6700, galactosemia 1:24,500. From July 2016 to March 2018 4065 children were tested by the high-risk screening. 41 cases of IMD: MMA (*n* = 4), PA (*n* = 2), BTD (*n* = 3), UCD (*n* = 4), GA I (*n* = 3), GA II (*n* = 2), MSUD (*n* = 4), NKH (*n* = 2), HCU (*n* = 4), LCHAD (*n* = 6), VLCAD (*n* = 1), TYR-I (*n* = 2), BKTD (*n* = 1), Mitochondrial diseases (*n* = 3) were detected. High-risk screening efficiency was 1:99. From January 2018 to March 2018 23600 newborns were screened for the IMD. We recalled 48 neonates (0.2%) with positive tests. 4 cases of PKU were diagnosed. In Moscow the frequency of PKU and Galactosemia is slightly higher than in European countries. The data from high-risk screening indicates the need of expanding the newborn screening in Moscow.

### P06. The Algorithm of Neonatal Screening for Phenylketonuria in Kazakhstan

SalimbayevaDamilyaScientific Center of Obstetrics, Gynecology and Perinatology, Republican Medical Genetics Center, Almaty 050012, Kazakhstan

One of the effective methods of the early detection and treatment to prevent mental retardation by phenylketonuria (PKU) is neonatal screening. In 2007 the national program of neonatal screening for PKU was established in Kazakhstan. The algorithm was based on international recommendations (ESPKU, ACMG, ISNS). Blood is taken from all newborns between 24 and 72 h after birth, bloodspot transport to regional screening departments and results of NBS should be ready after 3 days. If in the first bloodspot the Phe level is >120 µmol/L a retest should be done. Newborns with a positive NBS result should be invited to a regional screening department for knowledge and diagnostic procedures and early treatment. The treatment should be initiated before 21 days of life with Phe levels >360 µmol/L. All patient should undergo PAH genotyping. Before starting treatment, a 24-h loading test can be performed. This algorithm of neonatal screening for PKU is more useful and successful for Kazakhstan considering legal issues and national health policy. Perspectives of the newborn screening for PKU in Kazakhstan include differential diagnosis of BH4 deficiencies (analysis of urine pterines and DPHR activity).

### P07. The Newborns Screening in the Republic of Kazakhstan

KirikbayevaMeruyertSalimbayevaDamilyaScientific Center of Obstetrics, Gynecology and Perinatology, Republican Medical Genetics Center, Almaty 050012, Kazakhstan

Newborn dried blood spot screening for congenital hypothyroidism (CH) and phenylketonuria (PKU) in the Republic of Kazakhstan was established in 2007. It covers all the regions in Kazakhstan and is fully financed by the government. National centralized newborn screening system was created with regular monitoring and evaluation of the efficiency indicators. We have uniform certified equipment and reagents of Perkin-Elmer in all regional screening departments with mandatory external quality assurance of CDC, USA and use uniform NBS algorithm of ISNS. We organized, mandatory annual trainings with following certification of all regional NBS specialists. NBS coverage has increased from 52% in 2007 year to 86% in 2017 year. Since 2007 2.5 million newborns were screened with the relative incidence of: 1:7500 for congenital hypothyroidism and 1:24,000 for classic PKU. 105 PKU and 336 CH confirmed bloodspot cases since 2007 have received timely treatment, and do not show any signs of intellectual disability. In the Almaty region and Almaty city the pilot project for cystic fibrosis (CF) was started in 2017–2018 years with the algorithm–IRT, IRT, determine sweat chloride and CFTR genotyping. 13,300 newborns were screened, out of them 120 (0.90%) re-tests with IRT > 70 ng/mL. One newborn with CF was found with IRT concentration–252 ng/mL, the preliminary frequency of CF in Kazakhstan population is 1 per 13,300. Perspectives of the newborns screening in Kazakhstan are to increase NBS coverage and decrease cost, to expand the list of NBS conditions–cystic fibrosis, to improve a system quality assurance, including program evaluation, validity of testing systems, efficiency of follow-up and treatment.

### P08. Romanian National Registry for Newborn Screening of PKU and Hypothyroidism

MoldovanuFlorentinaNanuIoanaArdeleanuIoanaCismasuAlexandraNanuMichaelaNational Institute for Mother and Child Health, Pediatrics, Bucharest 1, Romania

The enrolment to the registry began in year 2010, has progressed and has been periodically improved up to present day. The registry has 4 levels of access: administrator, maternity (where the dry spot samples are collected), local health authorities (which monitors the collection of samples in the maternities), laboratories of the diagnosis and treatment monitoring units. The registry also contains a folder that refers to the individual children that were diagnosed with PKU or hypothyroidism and which allows the introduction by the monitoring physician of updated information regarding the periodic clinical and biological evaluation, as well as the therapeutic prescriptions. The paper presents data and graphs from the national registry related to PKU and hypothyroidism of the newborns screened during the year 2017 in Bucharest reference center. The analysis concerns topics like: the day of blood collection on dry spot in maternity, the periodicity of sample submissions to the reference laboratory in Bucharest, the duration from collection of samples to the registration at the reference laboratory, the time interval from registration in the laboratory to the release of test results, the time registered until families of children with positive screening test are contacted, the time registered until the blood serum test results for confirmation of diagnostic are released. Based on the analysis performed by the listed activities, weak points shall be revealed, and necessary actions will be proposed to follow to increase the efficiency of the newborn screening program. The exchange of experience with colleagues from screening programs of other countries will add a good value to the propositions of improvement. The Romanian National registry of hypothyroidism and PKU is becoming a powerful tool in the general management of the screening program, but also in the long-time monitoring of clinical evolution and treatment for the individual cases of children with diagnosed PKU or hypothyroidism.

### P09. Comparison between Perkin-Elmer Qsight^®^ 210MD and Waters^®^ Acquity^®^ TQD When Measuring Six-PLEX FIA-MS/MS Assay of ABG, ASM, GAA, GALC, GLA and IDUA Enzyme Activities

HeTaoTrometerJoePotierAnnaHaddockJordanYeungTsun AuBandhakaviSricharanDiPernaJamesSmithSaraVranishAlyssaMaharajhRudraTurku, Perkin-Elmer, 20101 Turku, Finland

The ability to measure ABG, ASM, GAA, GALC, GLA, and IDUA enzyme activities using a single 3.2 mm dried blood spot (DBS) via a flow injection analysis tandem mass spectrometer (FIA-MS/MS) method has been well established and presented on over the past few years. Typically, the FIA-MS/MS platform of choice has been a Waters^®^ TQD. Perkin-Elmer^®^ recently launched a new mass spectrometer, the QSight^®^ 210MD. The data presented herein compares a variety of samples analyzed on both platforms. Identical plates were derived from the same incubated plate to demonstrate variation in the platform while mitigating variation coming from the assay. Results from this study showed there to be similar precision, resolution and limits of quantitation across both platforms. Furthermore, it had faster sample-to-sample time, allowing one to analyze a 96-well plate in half the time of the Waters^®^ system.

### P10. Analytical Interferences: Routine Use of Chromatographic Separation for Reducing the Incidence of False Positive Results in Standard Amino Acids and Acylcarnitines Screening

TarcomnicuIsabelaBocanceaDanielaCajuTincutaGicaMaria IuliaSimionRodicaStambouliDanaeCytogenomic Medical Laboratory, Newborn Screening, Bucharest 1, Romania

Analytical interferences are common pitfalls in standard amino acids and acylcarnitines screening by flow injection (FIA) tandem mass spectrometry (MS). Without chromatographic separation, isobaric compounds or artefacts from the biological sample, reagents or labware increase the signal of some analytes, giving a high incidence of false positive results. In our laboratory, we have developed a supporting LC-MS/MS method to reduce the number of reported false positives. Concentrations were measured on a system composed by an Agilent binary pump and autosampler, connected to an AB Sciex 3200 triple quadrupole mass spectrometer. Dried blood spots were processed with the derivatized method (Chromsystems MassChrom kit). Standard newborn screening was performed by FIA. The chromatographic separation was carried out on a Poroshell 120 EC-C18 (50 mm × 4.6 mm, 2.7 μm) column, eluted in gradient, with a mobile phase consisting of water and acetonitrile, both containing 0.1% formic acid. Quantification was performed in multiple reaction monitoring mode. Full scan and production acquisitions were also carried out in a tentative to identify the interferences. Beside leucine or isovaleryl-carnitine, with well-described analytical interferences, other mainly affected analytes were arginine, ornithine, hexanoyl carnitine, glutaryl carnitine, succinyl carnitine, lauroyl carnitine, in some runs with 25% of the results above the limit. This was a randomly occurring issue (a few times per year). One or more additional high intensity peaks were observed in the false positive samples after injection on-column, even after repeated blood sample extraction. The concentrations had to be adjusted accordingly. These findings are helpful in the routine reporting of newborn screening results.

### P11. Determination of Reference Ranges for the Selected IEM and Congenital Disorders among the Neonates of South India

MoorkothSudheerMathewElizabeth MaryLewisLeslie EdwardRaoPragnaManipal Academy of Higher Education, Pharmaceutical Quality Assurance, Manipal, India

A tentative reference range has been determined for the conditions such as congenital hypothyroidism, congenital adrenal hyperplasia, galactosemia, glucose-6-phosphate dehydrogenase deficiency, biotinidase deficiency, phenylketonuria and cystic fibrosis. This determination is done from the data of a pilot newborn screening project conducted at Udupi district of South India. Results obtained for the screening of a total of 2000 newborn of 3 to 5 days of age were used for calculating the tentative reference range. The data acquired for biomarkers of congenital hypothyroidism and congenital adrenal hyperplasia does not follow normal distribution criteria and hence the median and inter quartile range was determined. The biomarkers of G-6PD deficiency, neonatal total galactose, cystic fibrosis and biotinidase deficiency follows normal distribution. Hence the mean and standard deviation were reported. The obtained results were 0.32 (1.25, 0.01) * µU/mL, 4.31 (7.62, 3.02) * nmol/L, 6.90 ± 2.15 ^#^ U/g Hb, 0.90 ± 0.80 ^#^ mg/dL, 4.82 ± 3.68 ^#^ mg/dL, 25.29 ± 23.48 ^#^ ng/mL, 171.20 ± 104.30 ^#^ Units respectively for neonatal TSH, 17-hydroxy progesterone, G-6PD, phenylalanine, total galactose, neonatal IRT and biotinidase levels. (* Median (Q3, Q1), ^#^ Mean ± 2SD).

### P12. Combined Tandem Mass Spectrometry (MS/MS) Screening Method for Biotindase Deficiency, Tyrosinemia Type I (HT1) and Sickle Cell Disease (SCD)

KleinJeannetteBroseAnnemarieFrömmelClaudiaLobitzStephanBlankensteinOliverTurnerCharlesDaltonR. NeilCharité Universitätsmedizin Berlin, Newborn Screening Laboratory, 10117 Berlin, Germany

Modern newborn screening uses a variety of analytical techniques, e.g., qualitative colorimetry, immunofluorescence, and MS/MS. With the increasing number of conditions screened, there is growing demand to save valuable patient material and optimize laboratory processes. We report the use of TMS to detect SCD, biotinidase deficiency and HT1 simultaneously. SpotOn Clinical Diagnostics developed a dried blood spot (DBS) TMS method for SCD screening, based on extraction and tryptic digestion of hemoglobin from the DBS and detection of disease specific peptides [1]. The protocol has been adapted, to simultaneously measure biotinidase and porphobilinogen (PBG) synthase activities. Initial incubation with biocytin and 5-aminolevulinic acid is followed by tryptic digestion. MS/MS analysis of Hb peptides and substrate/product pairs identifies SCD status and biotinidase and PBG synthase activities. The latter is inhibited by succinylacetone, the pathognomonic marker for HT1. Samples with a ratio of biotin/biocytin <30% of the daily mean were regarded as suspicious for biotinidase deficiency. Results correlate very well with the output from our standard colorimetric assay. Within a cohort of 30.000 samples 8 newborns with SCD and 1 with biotinidase deficiency were identified, demonstrating the potential of combining simultaneous SCD screening with enzyme testing on a single DBS (3 mm) by MS/MS. A similar approach for PBG synthase and comparison of the results with direct quantitation of succinylacetone is currently under investigation.

**Acknowledgments:** Financial support by the foundation “Kindness for Kids” is gratefully acknowledged.

1. Daniel, Y.A.; Turner, C.; Haynes, R.M.; Hunt, B.J.; Neil Dalton, R. Rapid and specific detection of clinically significant haemoglobinopathies using electrospray mass spectrometry-mass spectrometry. *BJH* **2005**, *130*, 635–643.

### P13. Striving towards a Better Predictive Value from the Dutch Newborn Screening Results

MaaseRoseBouvaMarelleRinaldoPieroVisserGepkeVeldenMonique de Sain-van derSchielenPeterThe National Institute for Public Health and the Environment (RVM), Center for Health Protection (GZB), NL-3720 BA Bilthoven, The Netherlands

The Dutch Newborn Screening (NBS) program screens for 19 disorders and in the coming years will be expanded to 31 disorders. Biochemical analysis of extracted whole blood, obtained by heel prick, is evaluated using cut-off values for clinically relevant analytes and/or analyte ratios. In 2016, 172,752 neonates (99.2% total eligible) were screened in The Netherlands: 597 were referred, 176 received a confirmed diagnosis of one of the NBS panel disorders, and 62 an inconclusive outcome (TNO report 060.23085/01.01). Overall, the Dutch NBS program has a positive predictive value (PPV) of 29%. A false positive rate (FPR) of 0.24% is of concern as a cause of long-term parental anxiety and additional costs associated with follow-up contact and testing. Of the current disorders, eleven are screened using tandem mass spectrometry (MS/MS) (PPV = 52%, FPR of 0.024%); these disorders are the focus of this study. Post-analytical interpretive tools made available by Collaborative Laboratory Integrated Reports (CLIR; https://clir.mayo.edu) replace conventional reference ranges and cut-of values with, respectively, simultaneous covariate-adjusted moving percentiles and a condition-specific degree of overlap for every marker, calculating a composite score reflecting the likelihood for a given disease. The score is evaluated using a database containing 2.7 million reference profiles and 28,127 true and false positive cases collated from 43 NBS programs (as of 31 March 2018). Method: MS/MS results from 10,000 screens were combined with four covariates (age at collection in hours, birth weight in grams, gestational age in weeks, and sex), anonymized and submitted to CLIR. For each dataset, conventional evaluation outcomes were compared with CLIR evaluation. Results: The impact on clinical reliability of incorporation of CLIR within the NBS evaluation protocol is reviewed. It is expected that the FP rate will be reduced without greater risk of FN events and the PPV will be improved. Legal, patient privacy and ethical aspects are considered.

### P14. Newborn Screening by Tandem Mass Spectrometry LC/MSMS: Derivatized Versus Non-Derivatized

KHNEISSERIssamSinclairGrahamSaint Joseph University, Newborn Screening Laboratory, Beirut 11-5076, Lebanon

Newborn screening (NBS) by LC/MSMS offer the possibility to detect a large spectrum of disease from one blood spot at a low cost. The technology is expanding to most NBS labs with both derivatized (butyl esters) and non-derivatized methods commonly used. The Saint Joseph university of Beirut has used a derivatized non-kit method since 2007. The latter has proven to be sensitive to heat during the derivatizing and drying steps leading to degradation of the acylcarnitine standards through de-acylation. From October 2017 till December 2017, we conducted a study to evaluate the impact of the derivatizing temperature on the degree of de-acylation using the D3-carnitine (D3-C0) as the outcome measure. Also, we will report some incidental findings due to under-drying on one of the amino acids. Theoretically, D3-C0 should not be found in the final solution as it is not present in human blood and the added free carnitine internal standard (IS) is D9-carnitine. Any D3-C0 found results from the de-acylation of D3-labelled acylcarnitine IS’s given that the label is present on the carnitine fraction of the added acylcarnitine IS’s. At the baseline of heating temperature of our method (65 C), D3-C0 was found in the amount of 3 µM. We proceeded to increase the temperature by three 10 C intervals, we found that D3-C0 concentration increased each time by 1 µM, averaging around 6 µM at 95 C. Regarding the amino acids, we did find that drying temperature and conditions may affect the stability of amino acid IS’s. Incidentally, it was found that the intensity of the leucine IS reduced with temperature. However, this affect was not seen with the non-labelled amino acid, leading to an apparent increased concentration for leucine and the other amino acids that used labelled leucine as an IS. Based on the above, we decide to switch to a non-derivatized kit. The outcome has been better than expected with more positive outcomes including less corrosion of the equipment by avoiding butanoic-HCl, using less acetonitrile solution and the possibility to add many parameters for more diseases in the near future.

### P15. Preliminary Application of Region 4 Stork (R4s) Project in Newborn Screening by Tandem Mass Spectrometry in China

XinwenHuangZhenzhenHuFangHongGulinQianTingZhangZhengyanZhaoDepartment of Genetics and Metabolism, Children’s Hospital, Zhejiang University School of Medicine, Hangzhou 310052, China; xinwenhuang@126.com

To investigate the feasibility of Region 4 Stork (R4S) project used for newborn screening by tandem mass spectrometry (MS/MS) in China. This retrospective study was performed among 362,822 neonates screened by MS/MS from May 2015 to April 2016 in Zhejiang newborn screening center (ZNSC). Infants were grouped by screening result category. Raw data was uploaded into R4S website to perform post-analytical interpretive tools, then results were excluded with interpretation rules. The comparisons of normal population percentiles were done at five selected percentiles between ZNSC and R4S project. Compared with cut-off system by using R4S project, the positive predictive value and specificity increased from 3.7 to 8.3% and 99.40 to 99.75% respectively, and the false positive rate declined from 0.6 to 0.2%. Among 83 true positive group, two hyperprolinemia cases were missed and one β-ketothiolase deficiency case was misdiagnosis. 311,638 cases in true negative group were resolved by post-analytical interpretive tools, and the residual 48,916 cases were excluded with interpretation rules. In false positive group, 2185 cases were reduced to 897 cases. Results of percentiles comparison showed that levels of some markers were significantly different between ZNSC and R4S project. R4S project improved the newborn screening performance effectively, whereas leaded to misdiagnosis and missed diagnosis. Besides, many true negative cases should be excluded with interpretation rules. Optimization should be achieved based on Chinese normal population.

### P16. Screening and Diagnosis Model for Neonatal Inherited Metabolic Disease by Tandem Mass Spectrometry

HuiZouJinan Newborn Screening Center, Jinan Maternal and Child Care Hospital, Jinan 250001, China; zouhui819@163.com

Definite diagnosis processes explore treatments of inherited metabolic diseases screened by MS/MS. Through epidemiological and genetic analysis, we study the disease incidence, clinical characteristics and intervention means of inherited metabolic diseases in this region. From December 2010 to December 2015, in accordance with the principle of informed consent, Jinan newborn screening center used MS/MS technology to screen newborns’ blood samples. Screening diseases include 37 kinds of amino acids, organic acids, fatty acids and metabolic diseases, as well as the calculation of single disease and positive rates of disease classification screening. We collect clinical high-risk children and suspected inherited metabolic disease children at the same period, clarify the diagnosis by MS/MS, calculate the incidence of diseases diagnosed, and compare with the newborn screening sample spectrum and incidence. Through MS/MS platforms, we screened 61,702 cases of newborn, 1170 positive cases, positive rate of 1.8%, and diagnosed 40 cases of positive infants. The positive rate of diagnosis screening is 1:1542 (0.65‰), which contains 23 cases of 2 kinds of organic acid metabolic diseases, 10 cases of 3 kinds of amino acid metabolic diseases, 8 cases of 3 kinds of fatty acid metabolism diseases. The most diagnosed diseases with high-risk are methylmalonic academia and hyper-phenylalaninemia. It is necessary to screen neonatal babies generally in Jinan due to the high incidence of genetic and metabolic diseases. We have confirmed that the maximum incidence and the most harmful diseases in this local are methylmalonic academia and hyperphenylalaninemia.

### P17. Evaluation of Urine Impregnated in Filter Paper for Organic Acids Analysis by Gas Chromatography/Mass Spectrometry (CG/MS)

SepúlvedaMaria del Rosario TorresRiveraMarcelo Raúl RodríguezGarzaLaura Elia MartínezMedicine School, Genetics, Monterrey Nuevo León 64640, Mexico

The profiling urinary organic acids by CG/MS is an invaluable tool for diagnosing of several organic acidurias. However, the obtaining and sending liquid urine sample is not always easy, especially in Mexico where it is difficult to transport it to other city or state. Several works reporting the use of filter paper collection of urine samples for detecting pathologies as methylmalonic aciduria, glutaric, isovaleric, by CG/MS, with good results. The aim of this study was to evaluate a method for the extraction and detection of multiple organic acids, in urine samples soaked on filter paper. Liquid Urine samples of normal cases and with known organic aciduria were impregnated on filter paper Whatman 903, extracted according to established method and analyzed by CG/MS. The extraction efficiency and stability of standard markers were tested, and compared between liquid and urine on filter paper. Some samples in filter paper were left at room temperature by several days and others at 40 degrees, to test the temperature effect. The urine samples impregnated in filter paper showed a similar chromatogram, peak detection and intensity as the liquid samples, and they were still stable without significant degradation when they were exposed to room temperature and 40 degrees. The specific organic acids obtained from known organic aciduria patients were easily detectable and enough to make the diagnosis. The filter papers soaked with urines obtained from normal or patients with suspicious metabolic diseases are adequate for analysis of organic acidurias by CG/MS. The urine delivery to the laboratory can be handled more easily with this method and even could be used for newborn screening.

### P18. Validation of the Measurement of Amino Acids in Plasma, Urine and CSF Using ATRAQ Kit by Liquid Chromatograph/Tandem Mass Spectrometry

TahtamouniMontherMitriRolaSkrinskaVictor A.RamaswamyMamathaHamad Medical Corporation, Laboratory, Doha 3050, Qatar

Liquid chromatography (LC) methods for measurement of amino acids in body fluids range from post column derivatization with absorbance detection to pre-column derivatization with mass spectrometry (MS) detection. Amino acid analysis by LC/MS is a rapid method and is reported to have the least interference from co-elution metabolites. This report describes the validation of the measurement of amino acids using the aTRAQ kit on a QTRAP 4500 ABSciex LC/MS analyzer including a comparison with the MASSTRAK ultra performance liquid chromatography (UPLC) method. The method summarized here is a modification of the published aTRAQ method. Norleucine was selected as the internal standard for quantitation of acidic and basic amino acids including alloisoleucine and argininosuccinic acid. The derivatized stable isotope standards were used to verify peak identity in the samples. The norleucine internal standard was added to each sample before extraction and derivatization. Thus, any loss of sample during extraction and derivatization was compensated by the internal standard. Calibrations for basic and acidic amino acids were linear from 2.5 to 1000 µmol/L and the detection limit was <2.5 µmol/L. The average coefficient of variation was 3.98% for 20 within day measurements. Correlation between two QTRAP 4500 instruments had a slope of 0.97 and an r2 of 0.95. Correlation between the QTRAP 4500 and the MASSTRAK UPLC had a slope of 0.89 and an r2 of 0.91. The MultiQuant software was used instead of Cliquid to allow direct calculation of analyte concentrations based on the norleucine internal standard. The method describing in this report provides a sensitive, accurate, and reproducible measurement of amino acids with a rapid analysis time of 20 min. The method is suitable for routine measurement of amino acids, including alloisoleucine and argininosuccinic acid, in body fluids.

### P19. Newborn Screening for MCAD Deficiency: Experience of the First 8 Years in Eastern Andalusia, Spain

Castro-VegaIsabelJiménez-MachadoRocíoSerrano-NietoJulianaBlasco-AlonsoJavierBenitoCarmenPérez-CerdáCeliaYahyaouiRaquelMálaga Regional University Hospital, Laboratory of Metabolic Disorders and Newborn Screening Center of Eastern Andalu, Málaga 29010, Spain

Medium-Chain Acyl-CoA Dehydrogenase Deficiency (MCADD) is an autosomal recessive fatty acid oxidation disorder with a potential fatal outcome. It is caused by mutations in the ACADM gene; the most prevalent mutation is c.985A > G. Evaluate the prevalence, clinical course, and biochemical and molecular phenotype of MCADD cases detected in the first 8 years of newborn screening in our center. From April 2010 to March 2018, the acylcarnitine profile, including C6, C8, C10, and C10:1, of 341,152 newborn DBS cards was measured by tandem mass spectrometry. Newborns with screen positive results were referred to physicians for further confirmatory testing (plasma acylcarnitine analysis and identification of biallelic pathogenic variants in the ACADM gene) and follow-up care. 21 newborns were referred for confirmatory testing for having C8 values above the screening cut-off of 0.17 µmol/L. They had a mean level of 5.83 µmol/L (range 0.37–32.38). All 21 had elevated C6 levels and 15 also had elevated C10 values. 20 of the screen positive infants had an MCADD diagnosis confirmed by plasma acylcarnitine analysis. Molecular testing was available for 12 confirmed cases: 6 were homozygous for the common c.985A > G mutation, 2 were compound heterozygous for c.985A > G, and 4 had other mutations. The average follow-up period was 3.5 years. One patient was lost to follow-up during the first year. Two patients had a metabolic crisis; both were homozygous for the c.985A > G mutation. The estimated prevalence of MCADD is 1:17,157 live births. MCADD frequency in our center is comparable to reports from other newborn screening programs. Early detection and treatment have successfully prevented adverse health outcomes in our MCADD patients.

### P20. Second-Tier Test in the First DBS in Newborn Screening of Methylmalonic Aciduria, Propionic Acidemia and Homocystinurias: Catalonia Experience

GarcíaSonia PajaresSastreAleix NavarroGaleraRosa Maria LópezSoriaJose Luís MarínRamírezAna ArgudoJiménezJose Eduardo FloresCarreiraCarmen MartínezAmoJose Antonio ArranzRieraMireia Del ToroIriberriRafa ArtuchHerreroAida OrmazabalGarcía-CazorlaAngelsOlivasSilvia MeavillaCastejónEduardoBordónRosa Maria FernándezRubióAntonia RibesHospital Clinic. Barcelona, Section of Inborn Errors of Metabolism. Biochemistry and Molecular Genetics Dep, Barcelona 08036, Spain

Methylmalonic and propionic acidurias, as well as homocystinuria, are an extensive group of autosomal recessive diseases included in the expanded newborn screening (NBS) program in several countries. Their detection can be performed through the analysis of primary markers [propionylcarnitine (C3), propionylcarnitine/acetylcarnitine ratio (C3/C2), C3/methionine ratio (C3/Met), Met or heptadecanoilcarnitine (C17)] On dried blood spots (DBS). However, specificity and positive predictive value (PPV) is highly improved by the measurement of methylmalonic acid (MMA), total homocysteine (HCY) and methylcitric acid (MCA) in DBS as second-tier test. Our study included 207,310 newborns analyzed from 2015 to 2017. Second-tier-tests were performed on the first DBS in 7748 newborns that had alterations in primary markers above described. MMA, HCY and MCA concentration were normal in 80.5% (6241 cases) and, in 17% (1328 cases) a second sample was requested. Among these, 109 cases were directed to the clinical unit: 1 methylmalonic aciduria with homocystinuria (CblC), 1 methylmalonic aciduria type B (CblB), 3 methylmalonil-CoA mutase (MUT) deficiencies, 3 propionic acidemias and, 3 cystathionine-β-synthase (CBS) deficiencies were genetically diagnosed, besides 1 case of suspected MUT/CblA or CblB. Moreover, 87 maternal or newborn vitamin B12 deficiencies were also detected (44 confirmed and 43 suspected), and other 7 cases had biochemical alterations although vitamin B12 data was not available. Furthermore, 3 transient cases were also detected. In summary, our data strengthens the importance of second-tier tests on the first DBS. They increase specificity and PPV (80%), reduces number of unnecessary second requested samples, as well as, of children directed to the clinical unit to be further studied.

### P21. Newborn Screening and Follow-Up Results of Organic Aciduria in Zhejiang Province

FangHongXinwenHuangYuZhangJianbinYangFanTongHuaqingMaoXiaoleiHuangXuelianZhouRulaiYangZhengyanZhao*Department of Genetics and Metabolism, Children’s Hospital, Zhejiang University School of Medicine, Hangzhou 310052, China*Correspondence: zhaozy@zju.edu.cn (Z.Z.); springfall2012@qq.com

This study aims to investigate the prevalence of organic aciduria in newborns by MS/MS and follow-up results of the confirmed patients in Zhejiang province. Acylcarnitine spectrum of 1,861,262 newborns from Zhejiang province were detected with MS/MS from January 2009 to December 2016. All positive patients were confirmed with urine GC/MS or gene analysis. 92 patients were confirmed with organic aciduria with a prevalence of 1/20,200. Among them, 40 patients had MMA; 13 of them (32.5%) were of MMA simple type and 27 combined type (67.5%). Genetic analysis was done in 7 patients with MMA simple type (MUT type, *n* = 6; CblB type, *n* = 1) and 11 with combined type (CblC, 10; CblF, 1). 6 patients had PA with a prevalence of 1/310200, 7 IVA, 1/265,900, 6 GA-1, 1/310,200, 27 MCC, 1/68,900, 1 HMG, 1/1,861,300, 2 BKD, 1/960,600, and 3 BD/HCSD, 1/620,400. 31 patients had a disease onset at neonatal period (MMA 25, PA 4, IVA 1, and GA-11), and 15 at post-neonatal period (MMA 7, PA 1, HMG 1, MCC 1, IVA 1, GA-12, BKD 1 and HCSD 1). 33 patients had brain involvements or cranial imaging disorders (MMA 21, PA 4, IVA 2, GA-1 3, HMG 1, BKD 1, HCSD 1); 3 patients with MMA had kidney diseases or hemolytic uremic syndrome, and 3 had myocardial impairments (PA 2, GA-1 1). 20 patients died (MMA 13, PA 3, HMG 1, IVA 1, HMG1, BKD 1). The overall prevalence of organic aciduria is not low in Zhejiang province. MMA is the most common organic aciduria, followed by MCC. Two-thirds of the patients have combined MMA, and most of them are of CblC subtype. Except MCC, other types of organic aciduria may lead to metabolism decompensation, complications or even death.

### P22. Newborn Screening for MCAD Deficiency: Experience of the First 8 Years in Eastern Andalusia, Spain

Castro-VegaIsabelJiménez-MachadoRocíoSerrano-NietoJulianaBlasco-AlonsoJavierBenitoCarmenPérez-CerdáCeliaYahyaouiRaquelMálaga Regional University Hospital, Laboratory of Metabolic Disorders and Newborn Screening Center of Eastern Andalu, Málaga 08036, Spain

Medium-Chain Acyl-CoA Dehydrogenase Deficiency (MCADD) is an autosomal recessive fatty acid oxidation disorder with a potential fatal outcome. It is caused by mutations in the ACADM gene; the most prevalent mutation is c.985A > G. Evaluate the prevalence, clinical course, and biochemical and molecular phenotype of MCADD cases detected in the first 8 years of newborn screening in our center. From April 2010 to March 2018, the acylcarnitine profile, including C6, C8, C10, and C10:1, of 341,152 newborn DBS cards was measured by tandem mass spectrometry. Newborns with screen positive results were referred to physicians for further confirmatory testing (plasma acylcarnitine analysis and identification of biallelic pathogenic variants in the ACADM gene) and follow-up care. 21 newborns were referred for confirmatory testing for having C8 values above the screening cut-off of 0.17 µmol/L. They had a mean level of 5.83 µmol/L (range 0.37–32.38). All 21 had elevated C6 levels and 15 also had elevated C10 values. 20 of the screen positive infants had an MCADD diagnosis confirmed by plasma acylcarnitine analysis. Molecular testing was available for 12 confirmed cases: 6 were homozygous for the common c.985A > G mutation, 2 were compound heterozygous for c.985A > G, and 4 had other mutations. The average follow-up period was 3.5 years. One patient was lost to follow-up during the first year. Two patients had a metabolic crisis; both were homozygous for the c.985A > G mutation. The estimated prevalence of MCADD is 1:17,157 live births. MCADD frequency in our center is comparable to reports from other newborn screening programs. Early detection and treatment have successfully prevented adverse health outcomes in our MCADD patients.

### P23. Decreased Concentrations of Isovaleryl-Carnitine in Patients with Maple Syrup Urine Disease (MSUD)

FingerhutRalph^1^KnerrIna^2^MonavariAhmad^2^BorovickovaIngrid^3^RöschingerWulf^4^1University Children’s Hospital, Zürich 8032, Switzerland2NCIMD TSCUH Dublin 1, Ireland3Biochem Dep and NBS Lab, TSCUH Dublin 1, Ireland4Laboratory Becker & Colleagues, Munich 81671, Germany

Newborn screening (NBS) for MSUD is a special challenge since patients can metabolically decompensate rapidly without adequate treatment within the first two weeks of life. The sum of the isobaric amino acids leucine, isoleucine, hydroxyproline (Xle), and Val are used as primary markers. For the confirmation of a positive NBS for MSUD a second-tier UPLC method for the separation of allo-isoleucine is usually used. From 491 samples of MSUD patients under treatment, we additionally measured the concentration of isovaleryl-carnitine (C5) in dried blood spots (DBS). C5 in MSUD patients was 0.02 ± 0.01 µmol/L (mean ± SD), reference range in healthy newborns 0.04–0.49 µmol/L (0.1st–99.9th centile). C5 in 9 NBS samples from patients with confirmed MSUD was in the range of 0.01–0.06 µmol/L. From these samples we calculated additionally the ratios of Xle/Alanin/C5, and Val/Alanin/C5. The ratio Xle/Ala/C5 proofed to be the best indicator for MSUD, with the lowest value of the TPs being 7.3 times higher than the highest value of 168 FNs. Val/Ala/C5 was the second-best indicator, followed by Xle/Ala and Val/Ala, with the lowest TPs being 4.1, 3.0, and 1.4 times higher than the highest values from FNs, respectively. As a proof of concept, we are retrospectively evaluating 5 MSUD cases from Ireland and 12 from Bavaria.

### P24. Newborn Screening and Vitamin Disorders

ChrastinaPetrBártlJosefBártováPavlínaHodíkJakubJežováRadkaPaulováMarkétaPinkasováRenataPetránkováVladislavaVláškováHanaKošťálováEvaHrubáEvaJahnováHelenaJešinaPavelKožichViktorPeškováKarolínaHonzíkTomášGeneral University Hospital in Prague and 1st Faculty of Medicine, Charles University, Department of Pediatrics and Adolescent Medicine, Prague 12808, Czech Republic

In June 2016 the Czech national newborn screening program was expanded to include 15 inherited metabolic disorders (IMD). The program allows to detect directly biotinidase deficiency, and indirectly also some treatable B-vitamin deficiencies due to dietary restriction, malabsorption or an IMD in the child or the mother. Screening for MTHFR deficiency may detect patients with folate and vitamin B12 deficiency, while screening for MCAD deficiency may detect riboflavin and coenzyme Q deficiencies. The newborn blood spots were collected between 48 and 72 h after birth. Profile of butylated amino acids and acylcarnitines was analyzed by tandem mass spectrometry. Second-tier total homocysteine was measured after its reduction by LC-MS/MS method. Biotinidase activity was measured by a fluorometric enzyme assay. Between July 2016 and December 2017, we analyzed samples from 128,531 newborns. We detected 48 patients with IMDs. Disturbed metabolism of vitamins was detected in 9 patients with partial biotinidase deficiency (prevalence (P) 1:14,500) and in 3 patients with maternal vitamin B12 deficiency (P 1:44,000). We did not detect any newborn with riboflavin or coenzyme Q deficiency. The treatment with biotin or vitamin B12 prevented development of clinical symptoms in all patients. Addition of biotinidase activity and of low methionine with second-tier total homocysteine measurement led to increase of the detection rate from 1:3600 to 1:2700 and enabled an early and an efficient treatment of affected patients.

### P25. Carnitine Deficiency in Preterm Neonates Receiving Total Parenteral Nutrition: Experience from Newborn Screening in Qatar

RamaswamyMamathaSkrinskaVictor A.AbdohGhassan M.MitriRola F.JoshiRavi R.TahtamouniMonther M.AlabedMamoon A.Hamad Medical Corporation, Laboratory Medicine, Doha 3050, Qatar

Carnitine is an amino acid which plays an important role in the oxidation of long-chain fatty acids. Both infant formulas and breast milk contain carnitine. However, it is not routinely provided in parenteral nutrition solutions in Qatar. Preterm neonates have a reduced capacity to synthesize carnitine and may need supplementation if on long-term total parenteral nutrition (TPN). Newborn screening (NBS) in Qatar is performed on dried blood spots (DBS) collected at or after 36 h of birth. For preterm infants born before 32 weeks gestational age (GA), a successive DBS is collected at 32 weeks GA equivalent. This helps to identify metabolic disorders that may be missed on the initial card due to prematurity, and to diagnose carnitine deficiency related to TPN. The amino acid/acylcarnitine (AA/AC) profile on the DBS was obtained by tandem mass spectrometry analysis of the butyl esters. We collected data for the premature babies with AA/AC profile showing carnitine deficiency (low C0 acylcarnitine) on the initial and/or successive 32-week GA equivalent DBS, for the years 2016 and 2017. A total of 8.36% premature babies were diagnosed with carnitine deficiency related to TPN during this period. Of these 3.04% of the babies were diagnosed on the initial NBS and 5.32% on the successive 32-week NBS with first screen being normal. Neonates who were diagnosed with carnitine deficiency on the first DBS had gestational age at birth ranging between 23 weeks and 29 weeks, and birth weight between 620 g and 1090 g. Most of these babies had at least three low markers on the AA/AC profile (low C0, C2, C3, C16, C18, C18:1, Acs/Cit). These babies were supplemented with L-carnitine. Currently there is no evidence in the literature to support the routine supplementation of all parenterally fed premature babies with L-carnitine. Our practice of performing the NBS again at 32 weeks GA equivalent in extremely premature babies, helps to identify the ones which have carnitine deficiency secondary to TPN and supplement them with L-carnitine for optimal growth.

### P26. Molecular Screening of IN2G (c.293-13A/C > G) Mutation and Detected Genotypes among the Macedonian Patients with Classical form of 21-Hydroxylase Deficiency

AnastasovskaVioletaKocovaMirjanaPediatric Clinic, Genetic Laboratory, Department of Endocrinology and Genetics, Skopje 1000, Macedonia

Steroid 21-hydroxylase deficiency is present in 90–95% of all cases with congenital adrenal hyperplasia, an autosomal recessive disorder. Severe enzyme deficiency can present as a classical salt-wasting (SW) and simple virilizing form (SV). The In2G (c.293-13A/C > G) mutation in CYP21A2 gene, coding for 21-hydroxylase, abolishes enzyme activity on 0–1% of normal activity. It alters pre-mRNA splicing by activating another acceptor site for the splicing process and thus shifting the reading frame to create premature termination of translation. Fifty-seven DNA samples from Macedonian patients with clinical and laboratory signs of classical form of 21-hydroxylase deficiency, 25 SW and 32 SV, were collected and subjected to PCR/ACRS method for the detection of seven CYP21A2 point mutations (P30L, In2G, Del 8ntG110, I172N, V281L, Q318X and R356W). The patients were evaluated at the Department of Endocrinology and Genetics, University Pediatric Clinic, Skopje, Macedonia. Aberrant splicing mutation In2G (c.293-13A/C > G) was detected in 72% (18/25) of the SW patients on 66% (33/50) alleles, and in 40.6% (13/32) of the SV patients on 31.25% (20/64) of the alleles. The most prevalent was In2G/In2G genotype found in 15 (60%) SW and 7 (21.9%) SV patients. The other genotypes detected among SW patients were In2G/Q318X in 2 and In2G/V281L + Q318X + R356W in 1 patient. Among the SV patients were detected In2G/P30L genotype in 3, In2G/I172N in 1 patient, and two were heterozygotes for In2G with no detected mutation on the second allele. The In2G/In2G was the most prevalent genotype among the Macedonian patients with classical 21-hydroxylase deficiency. Our founding supports the role of the In2G mutation in classical phenotype of the disease.

**Keywords:** 21-hydroxylase deficiency; CYP21A2 gene; In2G mutation; genotype

### P27. Effect of Clinical and Environmental Factors on 17 Hydroxy Progesterone and Its Cut-off Values in Newborns-Insights from a Prospective Newborn Screening Project in Delhi State

KapoorSeemaVermaPrashantJainAshishPariPriyankaVarugheseBijoKumarSomeshPolipaliSunilVatsPallaviRamjiSiddharthThelmaB. K.Maulana Azad Medical College, Dept. Pediatrics, NEW DELHI 110002, India

To evaluate correlation between 17-OHP values and various clinical and environmental parameters, and to establish cut-off values for 17-OHP such that optimum sensitivity and lower recall is maintained in the Newborn screening program for congenital adrenal hyperplasia. Multicentric program including 20 state funded hospitals in the state of New Delhi, India from Nov 2014 to March 2017. Heel prick sample of 202,549 newborns were taken as a part of multicenter Newborn screening program. 17-OHP levels were determined on a Genomic Screening Processor by fluoroimmunoassay. Log transformation of 17-OHP values was done to test for effect of various variables (such as gestational age, birth weight, gender, age at sampling, type of feeding, season of birth) on 17-OHP values using linear regression and multivariable regression analysis. Three set of cut-off values, based on 17-OHP values in true positive cases and 99th percentile values of data were determined. Cut-off 1 was an absolute value 50 ng/mL for preterm and low birth weight infants and 37.5 ng/mL for full term infants. Cut-off 2 and 3 were based on gestational age and birthweight intervals. Recall rate, false positive rate and ROC curve were calculated for each set to test for accuracy of each. Gestational age, birth weight and postnatal age at sampling were significant negative predictors of 17-OHP values. All three cut-off values had reduced recall rates with cut-off 1 having the lowest. Area under ROC curve was maximum for gestational age-based interval cut-off. Cut-off with lower recall rates which will not compromise on overall accuracy should be preferred and used.

### P28. Wisconsin Screening Algorithm for Congenital Adrenal Hyperplasia: Addition of a Second-Tier Steroid Profile Assay

HeldPatriceBialkEricUniversity of Wisconsin, Wisconsin State Laboratory of Hygiene, Madison, WI 53706, USA

Newborn screening for congenital adrenal hyperplasia (CAH) has one of the highest false positive rates of any of the diseases on the Wisconsin panel. This is largely due to the first-tier immune assay cross reactivity and physiological induced changes in concentration of 17-hydroxprogesterone during the first few days of life To improve screening for CAH, Wisconsin developed a second-tier assay to quantify five different steroids (17-hydroxyprogesterone, 11-deoxycortisol, 21-deoxycortisol, androstenedione, and cortisol) by liquid chromatography tandem mass spectrometry (LC-MSMS) in dried blood spots, using previously published papers as reference. The method validation included testing of confirmed CAH patients, known false positives, and unaffected individuals. established its own reporting algorithm dependent upon birth weight that incorporates analyte concentrations, as well as two different ratios, to identify babies at risk for CAH. Using this data, the false positive rate for the CAH screening was reduced by 90%. Patients with both classic salt-wasting and simple virilizing CAH in addition to non-classical forms were identified. Additionally, the second-tier testing was implemented into the routine workflow that enabled timely reporting of abnormal results. This study replicates and expands upon previous published research, to implement a timely second-tier screening assay and reporting algorithm that substantially improves the positive predictive value for CAH. The next steps are to investigate additional modifications to the screening algorithm by incorporating time of specimen collection.

### P29. Newborn Screening for Pompe Disease: An Approach to the Surveillance of Late-Onset Patients

FiciciogluCanThe Children’s Hospital of Philadelphia, Perelman School of Medicine at the University of Pennsylvania, Pediatrics and Human Genetics, Philadelphia, PA 19104, USA

Pompe disease is an autosomal recessive disorder caused by deficiency of alpha-glucosidase (GAA). The result is lysosomal storage of glycogen that adversely affects the function of cardiac and skeletal muscle. Patients exhibit a broad variety of clinical presentations, including an infantile form of the disease that is manifest shortly after birth with severe cardiomyopathy. Patients with milder disease present later in life, even in adulthood, with skeletal muscle weakness. Enzyme replacement therapy (ERT) with recombinant GAA has become available for symptomatic patients. The availability of an effective therapy has prompted establishment of newborn screening programs that test for GAA activity in dried blood spots. In February 2016, the Commonwealth of Pennsylvania initiated mandatory screening for Pompe in all newborns. The program includes gene sequencing when GAA activity is low in blood. In the initial 25 months of the program a total of 25 patients with a positive screening test were referred to our center. Of these, 11 patients have been confirmed to have late-onset, and 1 patient has been diagnosed to have infantile onset Pompe. An additional 5 patients have persistently low enzyme levels and harbor one known pathogenic mutation and one VUS. The remaining 8 infants were carriers or harbor pseudodeficiency alleles. It is accepted that early initiation of ERT in symptomatic patients is associated with better outcomes. However, there are no formal guidelines regarding the monitoring of pre-symptomatic patients. Likewise, it remains unclear what laboratory values should trigger initiation of ERT in this population. We report here our experience with a multidisciplinary clinical program established to monitor pre-symptomatic, late-onset Pompe patients. Our program includes regular clinic visits combined with serial physical therapy evaluations and careful monitoring of the medical history, cardiac status and pertinent laboratory studies. Even at this early stage of development, we found that this approach has greatly informed our therapy initiation decisions.

### P30. Neonatal Screening for Lysosomal Disorders: A Single Center Experience in Italy

PoloGiuliaSalviatiLeonardoCazzorlaChiaraRubertLauraZizzoCarmelaDuroGiovanniDardisAndreaBembiBrunoZordanRobertaDesnickRobert J.BurlinaAlessandroUniversity Hospital Padua, Division of Inherited Metabolic Diseases, Padova 35122, Italy

The increasing availability of treatments and the importance of early intervention have stimulated newborn screening (NBS) for lysosomal storage diseases (LSDs). We present our experience screening newborns in North-East Italy to identify neonates with Mucopolysaccharidosis Type I (MPS-I) and Pompe, Fabry and Gaucher diseases. Activities of acid β-glucocerebrosidase (ABG; Gaucher), acid α-glucosidase (GAA; Pompe), acid α-galactosidase (GLA; Fabry), and acid α-L-iduronidase (IDUA; MPS-I) in dried blood spots (DBS) from all newborns during a 30-month period were determined by multiplexed tandem mass spectrometry (MS/MS) using the NeoLSD^®^ assay system. Enzymatic activity cut-off values were determined from 3.500 anonymous newborn DBS. In the screening study, samples were retested if the value was below cut-off and a second spot was requested, with referral for confirmatory testing and medical evaluation if a low value was obtained. From September 2015 to February 2018, 80.451 newborns were screened for the four LSDs. We recalled 90 neonates (0.11%) for collection of a second DBS. Low activity was confirmed in 42, who had confirmatory testing. 16/42 had pathogenic mutations: four Pompe, four Gaucher, six Fabry, and two MPS-I. The incidences of Pompe and Gaucher diseases were similar (1/20.113), with Fabry disease the most frequent (1/13.409) and MPS-I the rarest (1/40.226). The combined incidence of the four disorders was 1/5.028 births. Simultaneously determining multiple enzyme activities by MS/MS, with a focus on specific biochemical markers, successfully detected newborns with LSDs. The high incidence of these disorders supports this screening program.

### P31. Simultaneous Screening for Six Lysosomal Disorders Using DBS Samples and MSMS

KantolaJaanaMattssonPekkaSjöroosMinnaRaussiHanna-MariOllikkaPiaLindroosHanneVaahteraKatjaJaakkolaMarkkuPotierAnnaTrometerJoeCournoyerJasonPerkin-Elmer/Wallac Oy, Reagent Development, Turku 20101, Finland

The NeoLSDTM MSMS kit is a new IVD CE marked product for newborn screening. The NeoLSDTM assay is based on tandem mass spectrometry (“MSMS”) for the quantitative determination of the activity of six lysosomal enzymes, specifically ASM, GAA, GLA, ABG, GALC and IDUA, which are deficient in the lysosomal storage diseases Niemann-Pick A/B, Pompe, Fabry, Gaucher, Krabbe and MPS-I respectively. Activities of all six enzymes are determined from a single 3.2 mm punch from a dried blood spot (“DBS”). The specifically designed substrates and labelled internal standards are added onto each DBS sample via the reagent cocktail. Internal standards and the enzyme generated products are measured by Flow Injection Analysis–MSMS (“FIA-MSMS”) using multiple reaction monitoring (“MRM”) and the results are calculated and analyzed using MSMS workstation software. The NeoLSDTM assay is validated to be run with Waters^®^ Acquity^®^ TQD or Xevo^®^ TQD MSMS screening systems. The design validation studies for CE marking were conducted in Danish Statens Serum Institute (SSI, Copenhagen) and German Metabolic Laboratory and Newborn Screening Diagnostic Center, Hamburg University Medical Center (ICLD). Approximately 3000 newborn leftover samples were tested in both studies, 2000 in the population distribution phase and 1000 in the screening performance phase. Based on the population distribution data, the cut-off values were calculated and set to the 1st and 0.5th percentiles of the population activities and then applied in the screening performance phase. Screening performance studies were enriched with confirmed positive samples dispersed throughout the multiple assay runs: 28 positive samples in SSI and 53 in ICLD. All positive samples were identified using either one of the cut-off values, except one Fabry female with normal GLA level. False positive rate varied depending on the disease from 0.2% to 1.2% when 1st percentile was applied and from 0 to 0.8% when 0.5th percentile was applied.

### P32. Selective Screening for Pompe Disease on Dried Blood Spots Facilitates Earlier Diagnosis

BektasMehmet SelcukLiaoHsuan ChiehMemurSeymaKaracaMeryemChangYing ChenLiuMei YingTurkmezAkgun OlmezAlanHafize NurTanyalcinIbrahimTanyalcinTijenHsiaoKwang JenLokman Hekim Hospital, Pediatric Health and Disease Clinic, Van, Turkey

Because of the severity of Pompe disease (PD) and the availability of the treatment, there is a growing evidence of including PD in newborn screening battery. The aim of this work is to introduce a new element to improve the diagnosis of PD in newborns with low acid alfa-glucosidase (GAA) activity by using the analytical tool system published at the 13th International Congress of Inborn Errors of Metabolism-ICIEM 2017 poster#754; https://doi.org/10.1177/2326409817722292. For the diagnostic confirmation of infantile early-onset form pathology with cardiomegaly, we evaluated 3 enzymatic activity on dried blood spots matrix simultaneously: 1-GAA, 2-total GAA (MGA = maltase glucoamylase +GAA); 3-Neutral α glucosidase activity (NAG) with 4MU-alpha-D-glucopyranoside. Values were far above the threshold 1.8 where our unaffected baby distribution mean ± 2SD was between −0.16 and 1.66. Affected babies scores were between 1.86 and 11.95. The mutation analysis of the GAA gene confirmed the diagnosis, 2 of them were newborns with a score 9.99, was found to be c896T > C homozygote and c258dupC homozygote, the other was 1 year 2 months old with a score of 3.76 was found to be heterozygote C.-32-13T > C. Genetic mutation assays were performed by direct exon and exon/intron boundaries Sanger sequencing. A prompt diagnosis is achieved, and thanks to a close follow-up by our doctors for the treatment of the babies creating an efficient/effective integration of all parties participating in this diagnostic procedure; laboratory, medical staff, parents, and others. These results are evidence that DBS studies facilitate detection of patients with LDs. Our observation as a first stage aims to provide valuable information to be considered by the national newborn screening operators in Turkey to adapt the enzyme assays to fluorimetric micromethods or LC-MS technique, reducing the cost of reagents and mainly of the expensive substrates, by evaluating the operational issues, cost, false positive rate, and other aspects related to the inclusion of the Pompe test in the newborn screening program.

### P33. Analytical Scoring Tool Based on Enzyme Activity to Guide the Diagnosis of Pompe Disease Suspected Patients

TanyalcinTijenTanyalcinIbrahimTanyalcin Medical Laboratory, Selective Screening and Metabolism Unit, Izmir 35220, Turkey

Pompe disease is a storage disorder with a partial or complete deficiency of acid a-glucosidase (GAA). The identification of acarbose as one of the effective inhibitors of isoenzyme maltase glucoamylase (MGA) derived from neutrophils permits the assessment of GAA activity in blood samples, dried blood spots. 3 reactions were performed by fluorometric enzymatic assay: 1-GAA, was measured at pH 4.0 in the presence of inhibitor acarbose; 2-total GAA (MGA + GAA) measured at pH 4.0 without acarbose; and 3-Neutral a-glucosidase activity (NAG), measured at pH 7.3. The tGAA reflects the combined activity of the isoenzyme MGA and GAA and was measured to calculate the percentage of tGAA that was inhibited by acarbose by using the formula (tGAA − GAA)/tGAA. This study uses a unique scoring system based on GAA, NAG, and tGAA to guide the diagnosis of patients. The scoring system is composed of 10 classifiers, several of which were either taken or adapted from Chien et al., 2008. Out of these 10 classifiers, 9 of them are binary with values 0 or 1; the remaining is a floating-point classifier that ranges between-1 and 1. Overall, a given patients score can range between −1 and 10; effectively incorporates tGAA, NAA, GAA and percent inhibition into the decision process. The algorithm tested on 35 patients, 28 of whom were healthy. Based on these values a threshold with a score of 1.8 yields excellent diagnostic odds ratio, classifying all patients correctly. In Conclusion: Our scoring system provides separation between healthy and affected patients which cannot be explained by GAA, tGAA, NAG or inhibition percent alone. Additionally, this system provides basis for—development for more sophisticated algorithms that incorporates regression analysis—fine-tuning of the threshold using ROC curves based on dynamic patient databases; and in cases where diagnostic metrics show bimodal/trimodal distribution, extension of classifiers to incorporate ethnic background to increase reliability.

**Keywords:** Diagnostic tools; Lysosomal storage disease; Pompe disease; Glycogen storage disease type II; Acarbose

### P34. Rarescreen—A Cross-Border Newborn Screening Project Detecting SCID, Hemoglobinopathies and Familial Hypercholesterolemia

WinterTheresaGiżewskaMariaOłtarzewskiMariuszBlankensteinOliverKleinJeanettePacMałgorzataMüllerCorneliaOstrowskaIwonaDurdaKatarzynaNauckMatthiasUniversitätsmedizin Greifswald, Neugeborenenscreening-Labor, Greifswald 17475, Germany

Expanding the newborn screening panel and improving the medical care is of great interest in our region and was already realized in the past by implementing a state-wide screening for cystic fibrosis, which is now implemented nationwide in Germany. In the framework of a recently granted Interreg 5a EU project, the cross-border implementation of three additional newborn screening disorders is planned in the north-east of Germany (Mecklenburg-Vorpommern, Brandenburg) and in the north-west of Poland (Western Pomerania). The chosen disorders are of medical relevance and fulfill the criteria for newborn screening. Screening of severe combined immunodeficiency (SCID), hemoglobinopathies and Familial hypercholesterolemia (FH) will be implemented. Three newborn screening laboratories (Greifswald, Berlin and Szczecin) are involved, each implementing one of the three additional disorders and providing them for the other project partners. In the Szczecin screening lab, the SCID screening will be performed, quantifying TREC and KREC by qRT-PCR. The screening for hemoglobinopathies will be performed at the screening center of the Charité (Berlin) using tandem mass spectrometry. Tandem mass spectrometry will also be used for the screening of FH, which will be implemented in Greifswald. The realization of the additional screening offer will start in May 2018 and will uphold until the middle of 2020. A total of 100,000 newborns will benefit from the improved health prevention offer. All data and experience gained during this project will be considered for discussion concerning the perpetuation of these three additional screening disorders on the nationwide level.

### P35. First Newborn Screening Program of Severe Combined Immunodeficiencies in Spain

RamírezAna ArgudoSoriaJose Luís MarínGaleraRosa Maria LópezGarcíaSonia PajaresRosaYania Quintero de laBuzónTatiana ColladoSoler-PalacínPereMartín-NaldaAndreaFranco-JaravaClaraRubióAntonia RibesBordónRosa Maria FernándezHospital Clinic. Barcelona, Section of Inborn Errors of Metabolism. Biochemistry and Molecular Genetics, Barcelona 08306, Spain

Although severe combined immunodeficiencies (SCID) meet the criteria to be included in a neonatal screening (NBS) program, it remains a controversial issue due to the low observed prevalence in some countries. In January 2017, SCID NBS in Catalonia started, becoming the first Spanish region and European country to officially include it. The results obtained in the first 14 months of experience are evaluated. TREC quantification in dried blood spot (DBS 1.5 mm diameter) was performed with Enlite-NeonatalTREC kit of Perkin-Elmer. A diagnostic decision algorithm was taken from the literature, which was updated after a year of experience. The results obtained in the first 66,980 samples analyzed in 2017 are the following: retest rate 3.34%, rate of second samples requested 0.21%, rate of positive detections 0.02% (*n* = 14). In 2017, no cases of SCID were diagnosed. In 2018, according to our experience, the decision algorithm was updated (basically the retest cut-off was changed from 34 to 24 copies/µL) which led to a decrease of the retest rate from 3.34% to 0.69%. In addition, in the present year, the first case of SCID was detected and confirmed. Therefore, the prevalence of the disease in our population was 1:77,819. TREC quantification in DBS for SCID detection has been implemented satisfactorily in our NBS program. The retest and request rates for second samples were optimal, and close to those published by others. The number of children visited in the clinical unit has been very low. The only NB with SCID, TREC was not detected, confirming the validation of the measurement system. The results obtained consolidate our strategy through the updated diagnostic algorithm and the prevalence obtained in our population justifies the inclusion of this disease in our NBS program.

### P36. The Performance of a New, Homogenous Single-Point PCR Assay for Simultaneous Quantitation of TREC and KREC

VeikkolainenVilleMäkinenMaariKerokoskiPetriOllikkaPiaFrangHeiniSaloVernaHjortMikaelWottonTiffanyEsberRoslynWileyVeronicaRaussiHanna-MariPerkin-Elmer, R&D, Reagent Development, Turku 20101, Finland

The objective was to evaluate the performance of a PCR research assay developed for quantitative determination of T-cell receptor excision circle (TREC) and kappa-deleting recombination excision circle (KREC) DNA in blood specimens dried onto a filter paper. TREC is an established marker for severe combined immunodeficiency (SCID) screening in newborns. In healthy newborns, TRECs are made in large numbers, while in infants with severe combined immunodeficiency (SCID) TRECs are barely detectable. However further research is needed to characterize the potential role of KREC as a marker for various immunodeficiencies. The new EnLite™ TREC-KREC RUO kit (research use only) involves a simple pre-treatment of the sample, followed by single-point PCR for TREC, KREC, and a control gene beta-actin. Homogenous detection is used to quantitate the PCR products. The performance of the new research assay was studied using dried blood spot samples. The precision, analytical sensitivity, linearity, and normal newborn population distribution of TREC was similar to the existing EnLite™ Neonatal TREC kit (IVD). Similarly, for KREC, the precision, analytical sensitivity, and linearity studies showed that at the normal newborn population distribution range there was consistent KREC amplification, whereas analyte-free samples remained un-amplified. The studies suggest that the new EnLite™ TREC-KREC RUO kit offers a simple method to be used in characterization of the TREC and KREC levels in research samples. The first research dataset on the TREC and KREC levels in different types of research samples is to be presented in the meeting.

### P37. Screening of Inborn Genetic Disorders X-ALD, ADA-SCID, ASA-LD and OTCD with Specific New Analytes Included in the Neobase™ 2 Non-Derivatized MSMS Kit

AppelblomHeidiLehtonenTeroBrozinskiJenny-MariaDorleyChristineMcKinlayMargoPölönenTuukkaKoivuAkiVaahteraKatjaPerkinElmer, Turku 20101, Finland

In total 57 analytes can be screened with the CE marked next generation NeoBase™ 2 Non-derivatized MSMS kit, providing more conclusive screening results and possibility to screen more inborn errors of metabolism when compared to the previous non-derivatized MSMS kit, NeoBase™. New analytes include very long-chain (C20-C26) lysophospholipids and acylcarnitines, which levels reflect the abnormal very long-chain fatty acid (VLCFA) profiles in the X-linked adrenoleukodystrophy (X-ALD) patients. X-ALD occurs, when mutated ABCD1 gene encodes defected adrenoleukodystrophy protein (ALDP) involved in VLCFA transport and thus leading to VLCFA accumulation in body. Specific markers for Adenosine deaminase (ADA), adenosine and 2-deoxyadenosine, have been included to effectively screen ADA patients. Deficient ADA enzyme has reduced ability to convert deoxyadenosine to non-toxic metabolites causing eventually severe combined immunodeficiency (SCID). 10–15% of all SCID cases have ADA deficiency, which onset may be delayed. Additional specific markers, Argininosuccinic acid (ASA), Glutamine (Gln) and Glutamic acid (Glu) were included to improve screening performance of two of the most common Urea cycle disorders, Argininosuccinic acid lyase deficiency (ASA-LD) and Ornithine transcarbamylase deficiency (OTCD). Two external studies were conducted in US. In both studies approximately 2000 newborn specimens were tested for population distribution and 2500 specimens to demonstrate screening performance including 4 X-ALD, 4 ADA-SCID, 3 ASA-LD and 3 OTCD confirmed positive specimens. All listed specimens were identified, or screening performance was shown to be improved when NeoBase™ 2 assay with new analytes was used.

### P38. First Experience with the TREC/KREC-Assay “Screen-Id” from Immuno IVD

FingerhutRalphUniversity Children’s Hospital, Swiss Newborn Screening Laboratory and Children’s Research Center, CH-8032 Zürich, Switzerland

Background: Newborn screening (NBS) for SCID has been approved by the Swiss Ministry of Health (BAG) in 2018. During the application, it was decided that SCID screening in Switzerland should not only use T-cell receptor excision circles (TRECs) as the primary marker, but also kappa recombining excision circles (KRECs). However, before the official start in January 2019, we had to determine our cut-off values, and establish the determination of TRECS and KRECS with the approved test system in the NBS laboratory in Zürich. Results: The test system could easily set up in the routine NBS laboratory, since second-tier DNA testing for cystic fibrosis is already part of the Swiss NBS program since 2011. Necessary additional instrumentation was 2 Quantstudio qPCR systems, 2 additional Thermocyclers, and an additional centrifuge with swingout buckets for microtiter plates for the post-PCR area. We calculated our initial cut-offs for TREC and KREC copy numbers from 1500 normal newborn screening samples from term babies. Additionally, we checked the TREC and KREC copy numbers from 100 preterm babies taken at the 4th day of life and the 14th day of life. To proof sensitivity, 10 NBS samples from newborns with confirmed SCID were tested.

### P39. Reliability of a Multiplex QPCR Assay for the Newborn Screening of SCID, SMA and XLA

Gutierrez-MateoCristinaFilippovGalinaDallaireStephanieRichardsJoel W.WuRongcongPerkin-Elmer, Diagnostics, Waltham, MA 01862, USA

The Recommended Uniform Screening Panel (RUSP) is a list of disorders that are recommended by the Secretary of the Department of Health and Human Services (HHS) for states to screen as part of their state universal newborn screening (NBS) programs in the US. Disorders on the RUSP are chosen based on evidence that supports the potential net benefit of screening, the ability of states to screen for the disorder, and the availability of effective treatments. In 2010 SCID screening was added to the RUSP allowing affected infants to be identified and receive treatment sooner. Similarly, evidence supporting the benefits of universal newborn screening of SMA has been reviewed and the condition is likely to be added to the RUSP in 2018. We have developed a four-plex real-time PCR assay to screen for SCIDs, XLA and SMA in DNA extracted from a single 3.2 mm punch of a dried blood spot (DBS). A simple high-throughput buffer DNA extraction method was developed for a Janus liquid handler that can process 384 DBS punches in four 96-well plates in just over one hour. The PCR assay identifies the absence of exon 7 in the SMN1 gene while simultaneously evaluating the copy number of T-cell receptor excision circles (TREC) and Kappa-deleting recombination excision circles (KREC) molecules. Additionally, the amplification of a reference gene, RPP30, was included in the assay as a quality/quantity indicator of DNA isolated from the DBS. The assay performance was demonstrated on over 1000 DNA samples isolated from punches of putative normal newborn DBS. The reliability and analytical accuracy was further evaluated using DBS control and contrived positive samples. The results from this study demonstrate the potential of future molecular DBS assays and highlight how a multiplex assay could benefit newborn screening programs.

### P40. Referrals and Incidental Findings for Newborn Screening for SCID in the Netherlands: First Results of the SONNET Study

BlomMaartjeBrediusRobbertde VriesMartineDekkersEugenieSchielenPetervan der BurgMirjamNational Institute of Health and the Environment (RIVM) and Leiden University Medical Center (LUMC), 2333 ZA Leiden, The Netherlands

Newborn screening for severe combined immunodeficiency (SCID) is based on the detection of T-cell receptor excision circles (TREC) by PCR. Previous studies have shown that newborn screening for SCID is accompanied by several incidental findings. In some cases, these conditions are untreatable such as ataxia telangiectasia (AT), raising ethical discussions in the newborn screening field. From 1 April 2018, 70,000 newborns in the Netherlands will be screened for SCID as part of the SONNET study. We present the first results of this Dutch pilot study including referrals, incidental findings and ethical implications. Although newborn screening for SCID is fully implemented in the United States, most countries in Europe are conducting pilot studies, as PCR is a new, relatively expensive test method for screening laboratories. The prospective SONNET-implementation pilot focuses on the practical implications, test qualities, costs and perspectives of parents/health care providers. The cut-off value, number of referrals, incidental findings and ethical issues are of particular concern. During a one-year period, all newborns in three provinces of the Netherlands will be screened for SCID. With a commercially available SCID-screening assay, T-cell specific TRECs, B-cell specific KRECs and internal control beta-actin will be detected in dried blood spots by quantitative real-time PCR. Newborns with TREC-levels below cut-off (≤6 copies/µL) will be referred according to a pre-set follow-up protocol. The results of the first six months of the SONNET study indicate that a follow-up protocol for incidental findings of SCID-screening is of great importance. The decision to screen strictly for SCID or screen for T-lymphocytopenia has a direct influence on the TREC cut-off value. Ethical implications will be addressed extensively in the final stage of the pilot study in which (ELSI-) questionnaires will be developed to query parents and health care providers about newborn screening for SCID and the expansion of the newborn screening program.

### P41. Newborn Sickle Cell and Thalassaemia Screening Program: Automating and Enhancing the System to Evaluate the Screening Program

CoppingerCatherineSCT Screening Program, Public Health England, London SE1 6LH, UK

The first evaluation of newborn screening program established in England in 2006 shows promising outcomes and scope for improvement. Test performance and coverage appear excellent but timeliness of care, acceptance of penicillin and adherence are challenges. Delays between screening results and enrolment in care require optimization of fail-safe follow-up. The newborn outcome system, built on user needs, will automate the gathering and reporting of newborn outcome data collected on SCT screen positive infants, it aims to:

- improve patient safety by allowing users to view the status of patients along the care pathway, for example to enable a referring laboratory to see that a screen positive baby has entered care
- alert clinicians when important milestones are breached
- improve quality and completeness of data to evaluate the program
- reduce duplication of data entry—the system must integrate with the National Haemoglobinopathy Register and the National Congenital Anomalies and Rare Disorder Registration Service
- reduce manual chasing through automated prompts
- improve reporting to data providers by accessible pre-determined reports for example key performance indicators including baby’s age at results to parents, prescription of penicillin and enrolment into treatment

Working in partnership with suppliers Medical Data Systems and Solutions and using an Agile project management we are automating and enhancing the system.

### P42. The Reliability of the Current Newborn Screening Action Value for the Detection of Beta Thalassaemia Disease in England

DanielYvonneHenthornJoanPublic Health England, Sickle Cell & Thalassaemia Screening Program, London SE1 8UG, UK

Beta thalassemias are a group of hereditary red cell disorders resulting in a reduced or absent production of the main adult hemoglobin, HbA. In England the NHS Sickle Cell and Thalassaemia Screening Program detects and reports newborn beta thalassemia disease as an incidental finding when testing for sickle cell disease. The current action value to initiate further investigations is 1.5% HbA using high performance liquid chromatography or capillary electrophoresis. We report the results of a 44-month prospective study to examine the reliability of the action value. 81 cases were reported with a HbA of 1.5% or less at first line screen. 9 were lost to follow-up. There were 6 false positive results, all 32 weeks gestation or less. 66 were true positives, 36 with confirmatory molecular results (11 of these cases also have results from tandem mass spectrometry), 19 had clinical confirmation, 11 had the results of both parents available; consistent with the screening result. There was one false negative, a confirmed beta thalassemia major case which had a HbA% above the action value at first line screen (1.7%) but was known to be at risk from parental results. Screening protocols are designed to maximize the detection of true positive cases and minimize numbers of false positive and negative results. The action value of 1.5% HbA is at the limits of sensitivity for the methods in use and is impacted by the fetal to adult hemoglobin switch with premature babies known to give false positive results. This study demonstrates a positive predictive value of 91.7%, with a specificity of 99.9% and a sensitivity of 98.5% these results confirm the reliability of the current action value.

### P43. APHL Hemoglobinopathies Workgroup—A Model for Effective Partnership for Resource Development

YusufCareemaAssociation of Public Health Laboratories, Newborn Screening and Genetics, Silver Spring, MD 20910, USA

The APHL Hemoglobinopathies Workgroup is comprised of newborn screening (NBS) hemoglobinopathy laboratory leads, a follow-up supervisor and physicians representing US NBS programs, as well as federal partners. The workgroup focuses on improving practices surrounding screening, diagnosis, education and follow-up of individuals with sickle cell disease (SCD) and thalassemia. To describe the workgroup’s efforts in pooling their collective knowledge and resources as well as from other stakeholders to create effective tools and resources for preventing and reducing complications of SCD and thalassemia. The workgroup meets monthly via teleconference calls and in person annually where ideas are discussed and those that are selected for further action are further developed. Communication also occurs via email as needed and input is sought via national surveys and listservs from other stakeholders including other US NBS programs and clinical specialists. Results:

- A comprehensive document and webinar series that identify best practices in US NBS programs and regional sickle cell treatment centers. Topics covered include specimen collection tips, screening methodologies, interpretation of screening results, limitations, and quality assurance and proficiency testing activities.
- A webinar series that describes the status of alpha thalassemia screening in the US and details the complexities of detecting and reporting alpha thalassemia as well as the clinical aspects of the condition.

Conclusion: The APHL Hemoglobinopathies Workgroup is a collaborative effort that provides expertise to build and enhance screening and diagnostic capacity for hemoglobinopathies.

### P44. Newborn Screening of Haemoglobinopathies in Catalonia: Three Years of Experience

SoriaJose Luís MarínGarcíaSonia PajaresRamírezAna Argudode la RosaYania QuinteroBuzónTatiana ColladoPastorDavid BeneitezAlvarezAdoración BlancoRubióAntonia RibesBordónRosa Maria FernándezGaleraRosa Maria LópezHospital Clinic. Barcelona, Section of Inborn Errors of Metabolism. Biochemistry and Molecular Genetics Depa, 08306 Barcelona, Spain

Catalonia is the Spanish region (CCAA) with the largest African population and the highest number of newborns with African parents. Newborn screening for sickle cell disease allows the early detection of the disease, prophylactic measures and a decrease in its morbidity and mortality. In January of the 2015 we started the Newborn Screening for sickle cell disease (SCD). To know the prevalence of SCD and carriers of HbS, C, D and E. Between 2015 and 2017, we analyzed dried blood spot samples from all newborns using capillary electrophoresis (Capillarys2Neofast, Sebia). The validation of results was done using the software Phoresis (Sebia) and Nadons (Limit4). The total number of newborns studied was: 207,310. The prevalence for SCD was 1/3141 (*n* = 66): FS (*n* = 46), FSβTAL (*n* = 2) and FSC (*n* = 18) phenotypes, being the most prevalent the FS phenotype (1/4508). Regarding carriers the prevalence was 1/156 (*n* = 1366) for HbS, and, 1/618 (*n* = 335), 1/4146 (*n* = 50), 1/3290 (*n* = 63) for HbC, HbD, and HbE, respectively. The prevalence for the bThalassemia major was 1/69,103 (*n* = 3) and 1/103,655 (*n* = 2) for a thalassemia major. 1. The prevalence of SCD in Catalonia is 1/3,141 and 1/156 for HbS carriers. 2. SCD is the second most prevalent disease in our program.

### P45. Duchenne Muscular Dystrophy Newborn Screening: Evaluation of a New GSP^®^ Neonatal Creatine Kinase–MM Kit in the US Population

PolariHannaTimonenAnneMeriöLiisaLloyd-PuryearMicheleMäkinenPauliinaAirenneSariStroberJonathanMcDonaldCraigHenricsonErikDayJohn WestDouineEmilieNelsonStanley F.KorpimäkiTeemuPerkin-Elmer, Wallac Oy, Turku 20101, Finland

Duchenne muscular dystrophy (DMD) is an X-linked rare degenerative neuromuscular disorder causing serious progressive muscle loss and premature death. DMD is a universally fatal genetic disorder and DMD affects approximately one in 5000 boys born worldwide. Early identification through newborn screening (NBS) can allow earlier intervention and may add years to an individual’s life span. Creating an easy to use, sensitive, and cost-effective screening test for DMD is an essential step in creating an NBS program. The progressive breakdown of skeletal muscle cells releases creatine kinase muscle isozyme (CK-MM) to the circulation, making it possible to screen for DMD with this biomarker. GSP Neonatal Creatine Kinase–MM kit is a new IVD immunoassay for the automated GSP^®^ platform to screen newborns for DMD. The objectives of the study were to determine the screening performance of the GSP CK-MM kit, to assess the effect of newborn’s age on the CK-MM levels present in circulation and to compare the performance of the GSP CK-MM kit with an enzyme activity-based laboratory developed test (LDT). The specimens were NBS dried blood spot samples from the California Biobank, collected from 21 DMD affected and 700 presumed unaffected newborns. The study was executed in Perkin-Elmer Wallac Oy. The CK-MM concentrations measured from the DMD specimens were within a range that was fully above the range of the unaffected population. Thus, all DMD affected specimens were detected with the GSP CK-MM kit using a 99th cut-off, whereas the LDT method missed one DMD affected specimen. Since the CK-MM concentration was found to inversely correlate with the age of the newborn in non-DMD specimens, laboratories should take this into account when the cut-off is established.

### P46. Clinical Performance Evaluation of the GSP^®^ Neonatal Creatine Kinase–MM Kit for Duchenne Muscular Dystrophy (DMD) Newborn Screening

PolariHannaTimonenAnneHougaardDavidLaitalaVilleMeriöLiisaSkogstrandKristinAirenneSariKorpimäkiTeemuPerkin-Elmer, Wallac Oy, Turku 20101, Finland

Duchenne muscular dystrophy (DMD) is the most common muscular dystrophy among children affecting mostly males (1:5000) and caused by mutations in the dystrophin gene. With DMD, muscle specific creatine kinase isozyme CK-MM is released into bloodstream enabling screening for DMD with this biomarker. GSP Neonatal Creatine Kinase–MM kit (3311-0010) is a new fully automated assay based on DELFIA^®^ technology for the GSP platform. This assay is the first IVD kit bearing CE mark to screen newborns for DMD. The objectives of the clinical performance study were to produce newborn population distribution data using the GSP CK-MM assay, assess the usability of the GSP CK-MM kit and demonstrate that the DMD affected population can be distinguished from the normal population. The specimens were archived newborn screening dried blood spot samples from the Danish Newborn Screening Biobank collected from 16 DMD affected and approximately 3700 presumed unaffected newborns. The mean value for presumed unaffected specimens was 128 ng/mL and for DMD affected 2626 ng/mL. With 99.5th percentile cut-off, 15 of the DMD confirmed specimens were classified as screening positive and one specimen from an extremely preterm, very low birth weight newborn was classified as negative. With 99.5th percentile cut-off the positive agreement was 93.8% and negative agreement 99.7%. GSP CK-MM kit was found to meet user needs and to discriminate well between normal population and affected DMD cases. However, future DMD newborn screening programs might benefit from using a different screening algorithm for low birth weight and preterm newborns.

### P47. Initial Evaluation of Prospective and Parallel Assessments of Cystic Fibrosis Newborn Screening Protocols in Eastern Andalusia: IRT/IRT versus IRT/PAP

YahyaouiRaquelJiménez-MachadoRocíoCastro-VegaIsabelBlasco-AlonsoJavierCaro-AguileraPilarSerrano-NietoJulianaBenitoCarmenRuiz-CortésEduardoCamino-LeónRafaelPérez-ValeroVidalPérez-FríasJavierPérez-RuizEstelaMálaga Regional University Hospital, Laboratory of Metabolic Disorders and Newborn Screening Center of Eastern Andalu, 29010 Málaga, Spain

In June 2011, our region began CF newborn screening. It adopted the IRT/IRT protocol, which leads to a high rate of false positives. Evaluate preliminary results of a prospective, sequential, and parallel IRT/PAP strategy compared to our newborn screening center’s current IRT/IRT strategy. From June 2017 to March 2018, the IRT/IRT strategy with cut-offs of IRT1 ≥ 61 ng/dL and IRT2 ≥ 40 ng/dL was used to screen 28,750 newborns. In parallel, PAP was assayed using a MucoPAP-F kit (DYNABIO, Marseille) in newborns with IRT1 ≥ 50 ng/dL. When IRT1 = 50–60.9 ng/dL, 2.1 μg/L was set as a provisional PAP cut-off for requesting a second sample to evaluate if PAP measurement could improve sensitivity in this range of IRT1 levels. A PAP concentration above 3.8 μg/L when IRT1 ≥ 50 and IRT2 ≥ 35 was considered a positive screen. Newborns with positive screening results according to one or both protocols were referred for further confirmatory testing (sweat test and/or identification of biallelic pathogenic variants in the CFTR gene). 440 newborns had IRT1 levels above 50 ng/dL and a second DBS sample was requested for 302 newborns (1.05%). Of them, 8 were lost to follow-up due to early death (*n* = 5), parental refusal (*n* = 1), or wrong address (*n* = 2). CF was detected in 6 newborns. All of them would have been detected by both strategies as they had high PAP values (median 5.96 μg/L, range 2.93–96.2). A ROC analysis of PAP determined a sensitivity of 100% and specificity of 89.9% for the point 2.91 μg/L. Using this cut-off, the number of sweat tests would have been 33 instead of 31 with the IRT/IRT strategy. Sequential measurement of IRT/PAP provides good sensitivity and specificity and allows for more reliable and cost-effective CF-NBS than the IRT/IRT strategy.

### P48. Audit of Second Samples Required for Newborn Screening of Cystic Fibrosis

SmithSarahNHS Greater Glasgow and Clyde, Scottish Newborn Screening Laboratory, Glasgow G51 4TF, UK

Scotland follows a cystic fibrosis (CF) protocol that may require a second sample for IRT analysis. This sample should be taken between day 21 to day 28 of life, but ideally on day 21. The sample can be taken up to day 56 of life after which CF screening is not recommended. Audit all samples received for CF screening in 2017 that required a second sample. Second samples taken out with the recommended time were analyzed in detail to determine root cause. 63 babies screened for CF required a second sample for IRT measurement. 13 samples (21%) were taken after the recommended time, but before 56 days of life. Two of these samples had delayed first sampling for CF screening and so were excluded from analysis. Of the remaining 11 samples 9 of these (82%) were received in the last quarter of 2017. Detailed analysis of each case established that there were 4 reasons for late second samples and actions were undertaken to combat each. (1) Normal post was used to request the second sample. Policy was changed to ensure that only email was used to request repeat samples. (2) The request for a second sample was sent to the neonatal units but the baby had been discharged home. Neonatal liaison nurses will be utilized to ensure the repeat request is sent to the appropriate person. (3) Community Midwives misunderstood the requirement to take the sample at a certain time. Midwife education has been delivered. (4) Misinterpretation of reports from the screening lab. Reports have been clarified. Late second samples increased significantly in the last quarter of 2017. Measures have been put into place to address this. However, this prompted a review of the processing of late second samples. There is a lack of clear evidence around interpretation of late second samples for CF screening, therefore for a trial period Scotland will amend the UK CF policy and late second samples will be discussed with appropriate healthcare professionals and further clinical evaluation may be requested. This will depend on how late the sample was taken, what the IRT result is and what the current 99.5th centile is.

### P49. Analysis of Strategies with Immunoreactive Trypsin and Pancreatitis-Associated Protein for the Detection of Cystic Fibrosis. Application in the Catalonia Neonatal Screening Program

GaleraRosa Maria LópezRamírezAna ArgudoGarcíaSonia PajaresLópezGiovanna DelgadoJiménezJose Eduardo FloresMorenoEster RamónOrquínCelia BádenasTizzanoSilvia GatnerRoigMaria Colsde la CruzOscar AsensioBordónRosa Maria FernándezRubióAntonia RibesSoriaJose Luís MarínHospital Clinic. Barcelona, Section of Inborn Errors of Metabolism. Biochemistry and Molecular Genetics Depa, 08306 Barcelona, Spain

The detection of cystic fibrosis was implemented in our newborn screening (NBS) program in 1999 with a double sample strategy for immunoreactive trypsin (IRT1 + IRT2), positive predictive value of 4.12% (PPV). Recent studies show that combining IRT with pancreatitis-associated protein (PAP) increases specificity and reduces the false positive rate (FP). To study different strategies combining IRT with PAP; to propose the most appropriate strategy to incorporate PAP and its application in our NBS program. 64,722 samples were analyzed between January and November of 2016. In the period studied, the IRT1 + IRT2 strategy was maintained (IRT1 ≥ 60 ng/mL samples of 48 h and IRT2 ≥ 35 ng/mL samples between 21 and 30 days). PAP was performed in samples with IRT1 ≥ 50 ng/mL. In the analysis of the strategies, different cut-off points were applied for IRT and PAP based on the bibliography and our results of true positives, true negatives and false negatives. Sensitivity and specificity were calculated. 18 strategies were analyzed with IRT (*n* = 893) and IRT + PAP (*n* = 1839). The chosen combination was: [IRT1 ≥ 50–80 + PAP ≥ 1.95 + IRT2 ≥ 35] or [IRT1 ≥ 80–150 + PAP ≥ 1 + TIR2 ≥ 35] or [IRT1 ≥ 150 + IRT2 ≥ 35]. This strategy (*n* = 1033) has been applied for 6 months (October 2017–March 2018), with a reduction of 46% and 53% in the second samples and FP, respectively, compared with the strategy used to date. The results with the chosen strategy of IRT + PAP show that specificity and PPV increase, with respect to our previous strategy with only IRT. It also allows a significant reduction in the number of FP, resulting in a reduction in the number of affected families and a lower cost for the program.

### P50. Reliability of a Long-Established Newborn Screening Program for Cystic Fibrosis–Review of Late Diagnoses

HeatherNatashaByrnesCassJaksicMirjanaTateJanWebsterDianneAuckland City Hospital, LabPlus, Auckland AK1, New Zealand

Newborn screening for cystic fibrosis (CF) has been continuous in New Zealand (NZ) since 1983. This has followed a 2-step process, with some changes in methodology. IRT/IRT (immunoreactive trypsin) was used up until 1996 and IRT/limited panel of common genes from 1996-present. A positive CF screen requires an abnormal result in both steps, i.e., raised IRT followed by one or 2 common CF mutations found. All children with CF in the greater Auckland region (population 1.2 million) have full care at Starship Children’s Hospital. To determine the number of children with a late CF diagnosis and review why they were missed by newborn screening (NBS). A retrospective review was performed on all children diagnosed with CF and seen at the Starship Children’s Hospital CF clinic over the period 2003–2017. Between 2003 and 2017, 110 children with CF were followed through the clinic. 89 children with CF were picked up and diagnosed following a positive newborn screening test and 21 children had a late non-screening diagnosis of CF. Mean age at late CF diagnosis was 32 months (range 6 weeks to 13 years). 8 children had been born abroad; 7 in countries without CF-NBS, 1 was picked up by NBS but had an equivocal sweat test. 13 children had been born in NZ; 1 family had refused NBS, 9 were missed as their IRT was not in the top 1%, 2 had high IRT but unusual genes, and 1 was detected by NBS but had an equivocal sweat test. Even with an established newborn screening program in place it is important that clinicians remain vigilant about late diagnoses of CF. CF can be missed at all steps of the screening and diagnostic pathway, but the most common reason within our population was having been born in a country without CF-NBS.

### P51. Initial Evaluation of Prospective and Parallel Assessments of Cystic Fibrosis Newborn Screening Protocols in Eastern Andalusia: IRT/IRT versus IRT/PAP

MacíasRaquel Yahyaoui^1^,^2^MachadoRocío Jiménez^1^Castro-VegaIsabel^1^Blasco-AlonsoJavier^3^Caro-AguileraPilar^3^Serrano-NietoJuliana^3^LópezCarmen Benito^4^CortésEduardo Ruiz^5^LeónRafael Camino^6^ValeroVidal Pérez^1^FríasJavier Pérez^3^RuizEstela Pérez^3^,^4^1Laboratory of Metabolic Disorders and Newborn Screening Center of Eastern Andalusia, Málaga Regional University Hospital, 29010 Málaga, Spain2Institute of Biomedical Research in Málaga (IBIMA), 29010 Málaga, Spain3Department of Pediatrics, Málaga Regional University Hospital, 29010 Málaga, Spain4Department of Genetics, Málaga Regional University Hospital, 29010 Málaga, Spain5D.G. Salud Pública, Consejería de Salud, 41020 Sevilla, Spain6Plan Andaluz para la Atención a Personas con Enfermedades Raras (PAPER), 41018 Sevilla, Spain

In June 2011, our region began cystic fibrosis (CF) newborn screening. It adopted the immunoreactive trypsin (IRT)/IRT protocol, which leads to a high rate of false positives. Evaluate preliminary results of a prospective, sequential, and parallel IRT/pancreatitis-associated protein (PAP) strategy compared to our newborn screening center’s current IRT/IRT strategy. From June 2017 to March 2018, the IRT/IRT strategy with cut-offs of IRT1 ≥ 61 ng/dL WB and IRT2 ≥ 40 ng/dL WB was used to screen 28,750 newborns. Specimen collection for IRT1 was done within 48–72 h after birth and within 24–28 days of life for IRT2. In parallel, PAP was assayed using a MucoPAP-F kit (DYNABIO, Marseille) in newborns with IRT1 ≥ 50 ng/dL WB. When IRT1 = 50–60.9 ng/dL WB, 2.1 μg/L WB was set as a provisional PAP cut-off for requesting a second sample to evaluate if PAP measurement could improve sensitivity in this range of IRT1 levels. A PAP concentration above 3.8 μg/L WB when IRT1 ≥ 50 and IRT2 ≥ 35 was considered a positive screen. Newborns with positive screening results according to one or both protocols were referred for further confirmatory testing (sweat test and/or identification of biallelic pathogenic variants in the *CFTR* gene). 440 newborns had IRT1 levels above 50 ng/dL WB and a second DBS sample was requested for 302 newborns (1.05%). Of them, 8 were lost to follow-up due to early death (*n* = 5), parental refusal (*n* = 1), or wrong address (*n* = 2). CF was detected in 6 newborns. All of them would have been detected by both strategies as they had high PAP values (median 5.96 μg/L WB, range 2.93–96.2). A ROC analysis of PAP determined a sensitivity of 100% and specificity of 89.9% for the point 2.91 μg/L WB. Using this cut-off, the number of sweat tests would have been 33 instead of 31 with the IRT/IRT strategy. Sequential measurement of IRT/PAP provides good sensitivity and specificity and allows for more reliable and cost-effective CF-NBS than the IRT/IRT strategy.

### P52. Stability of IRT in Dried Blood Samples

FingerhutRalph^1^NennstielUta^2^1Swiss Newborn Screening Laboratory, and Children’s Research Center, University Children’s Hospital, 8032 Zürich, Switzerland2Bavarian Health and Food Safety Authority, Screening Center, 85764 Oberschleissheim, Germany

Cystic fibrosis (CF) is one of the most common autosomal recessive disorders with a frequency of about 1 in 3500 livebirths in the white population. Switzerland started CF-NBS in January 2011, and Germany in September 2016. Due to an amendment to the act for Genetic Testing in Humans from 2010, the situation for the German NBS programs became very complicated. Due to the new legal regulations, the testing for CF can only be performed after the parents have been informed by a physician about CF-NBS. This does not only affect the second-tier mutation analysis, but also the primary IRT testing. IRT measurement must be postponed until this information reaches the laboratory. However, there is so far no information available on the short-term stability of IRT in DBS at ambient temperatures, which makes it rather impossible for NBS laboratories to define correct cut-off values for IRT, which is not immediately measured at the day of arrival in the NBS laboratory. We have confirmed the short-term stability of IRT in 42 leftover DBS. In addition, we also tested the variability of IRT measurements in 500 DBS where the initial IRT was >50 ng/mL whole blood.

### P53. Including Classical Galactosaemia in the Expanded Newborn Screening Panel Using Tandem Mass Spectrometry for Galactose-1-Phosphate

CohenAriehBaurekMartaLundAllanDunøMortenHougaardDavidStatens Serum Institut, Danish Center for Newborn Screening, 2300 Copenhagen, Denmark

Galactosaemia has been included in various newborn screening programs since 1963. Several methods are used for screening; however, the predominant methods used today are based on the determination of either galactose-1-phosphate uridyltransferase (GALT) activity or the concentration of total galactose. These methods can be used on their own or in combination in a two-tier regime. These methods cannot be multiplexed and therefore require one full punch per sample. Since the introduction of mass spectrometry in newborn screening, many diseases have been included into newborn screening programs because this technique allows the analyses to be multiplexed. Here we present a new method for including classical galactosemia in an expanded newborn screening panel with several clear advantages compared to existing approaches. We also demonstrate that incorporating galactosemia in the expanded newborn screening panel using tandem mass spectrometry, does not affect the screening performance for other metabolic disorders. The novel method does not call for an extra punch, the existing workflow only needs minor adjustments and it can be run on tandem mass spectrometers in routine use. Furthermore, compared with previously used methods, the present method has a superior screening performance producing significantly fewer false positive results. We present data from 5500 routine newborn screening samples from the Danish Neonatal Screening Biobank. The cohort was enriched by including 12 confirmed galactosemia positive samples and 10 samples that were positive for other diseases diagnosed through the Danish newborn screening program. All galactosemia positive samples could be identified by the method with no false positives. The cohort was also analyzed for GALT enzyme activity and we present a comparison between the two methods.

### P54. Recommendations for Newborn Screening for Galactokinase Deficiency: A Systematic Review and Evaluation of Dutch Newborn Screening Data

StroekKevinBouvaMarelleSchielenPeterVazFrédéricHeijboerAnnemiekeJongeRobert deBoschAnnetBoelenAnitaAcademic Medical Center, Department of Clinical Chemistry, Laboratory of Endocrinology, 1105 AZ Amsterdam, The Netherlands

Galactokinase (GALK) deficiency causes cataract leading to severe developmental consequences unless treated early. Because of the easy prevention and rapid reversibility of cataract with treatment, the Dutch Health Council advised to include GALK deficiency in the Dutch Newborn Screening program. The aim of this study is to establish the optimal screening method and cut-off value (COV) for GALK deficiency screening by performing a systematic review of screening strategies and total galactose (TGAL) values and by evaluating TGAL values of screened newborns in the Netherlands. Systematic literature search strategies were developed and study selection, data collection and analyses were performed by two independent investigators. TGAL values measured in the first week of life by the Quantase Neonatal Total Galactose screening assay in a cohort of Dutch newborns in 2007 were evaluated. Four studies describing screening strategies used TGAL as the primary screening marker combined with galactose-1-phosphate uridyltransferase (GALT) measurement that is used for classical galactosemia screening. TGAL COVs of 2200, 1665 and 1110 µmol/L blood resulted in positive predictive values (PPV) of 100%, 82% and 10% respectively. TGAL values measured in the newborn period were reported for 39 GALK deficiency patients with individual values ranging from 3963 to 8159 µmol/L blood and 2 group values with mean 8892 and 4856 µmol/L blood. Dutch Newborn Screening data of 72,786 newborns from 2007 provided a median TGAL value of 110 µmol/L blood with a range of 30–2431 µmol/L blood. Based on TGAL values measured in GALK deficiency patients reported in the literature and TGAL values in the Dutch Newborn Screening cohort we suggest performing GALK screening with TGAL as a primary marker with a COV of 2500 µmol/L blood, combined with GALT enzyme activity measurement as used in the classical galactosemia screening. This will ensure detection of GALK deficiency patients and minimize false positive referrals.

### P55. Clinico-Molecular Characterization of Glucose-6-Phosphate Dehydrogenase (G6PD) Deficiency in Indian Neonates

KapoorSeemaDeswalPreetiBhattacharyaUpasnaVarugheseBijoKumarSomeshPolipaliSunilJainAshishRamjiSiddharthThelmaB. K.Maulana Azad Medical College, Dept Pediatrics, New Delhi 110002, India

G6PD deficiency is the most prevalent form of metabolic disorder affecting 400 million people worldwide. Prevalence in India is 8.5% and is considered to be the highest worldwide. Higher prevalence is seen in north India as compared to south India. The aim of our study was to assess the clinical profile and perform molecular characterization of G6PD deficiency in north Indian neonates. A total of 9600 neonates were screened, out of which 40 were selected based on G6PD quantitative testing by spectrophotometric analysis using RBC lysate. Neonates found G6PD deficient were recalled at 48 h and 7 days of life to look for development of hyperbilirubinemia, peak serum bilirubin levels, requirement of phototherapy and exchange transfusion. For those found deficient, mutation screening was done based on prevalence of variant in the population. PCR-RFLP was done for G6PD Mediterranean (exon 6) and G6PD Orissa (Exon 3). Sanger sequencing was done for exon 9 and other uncommon mutations. 25 (62.5%) neonates developed jaundice, out of which 9 required intervention–6 babies underwent phototherapy and 3 required exchange transfusion along with phototherapy. 19 out of 40 i.e., 47.5% were found to be positive for G6PD Mediterranean, 16 (40%) were positive for G6PD Orissa, 2 (5%) for G6PD Mahidol and 1 (2.5%) each for Chattam, Kerala and Jammu. Most common mutation found was Mediterranean, 42% of the kids developed jaundice but did not require any intervention, 21% required intervention and rest did not develop jaundice. Second most common mutation was Orissa, 43.7% developed jaundice without requiring any treatment, 15.7% required intervention and rest were asymptomatic. One case of Kerala mutation required exchange transfusion, 1 case of Chatham required no intervention. To our knowledge, this is the first study with molecular aspects of the G6PD deficiency in neonates in India. This study emphasizes that G6PD is not a benign enzymopathy in India. G6PD screening at birth can help in triaging the neonates at discharge for timely follow-up.

### P56. NBS LIMS Support for Hearing and Critical Congenital Heart Disease Screening at Birth

CarterAnthonyHarmsVolkerIntegrated Software Solutions Ltd., Laboratory Information Systems, Winchester SO23 9EH, UK

This poster will describe how a Newborn Screening LIMS can support and enhance typically non-laboratory screening services. In this instance CCHD and Hearing with examples from a North American site. The LIMS allows for flexible data entry and customization of forms, automated rules to reduce both user input requirements and error while flagging suspect screening results. In addition, the system can provide for quick and easy reporting of results, integrate the clinical case management workflow and proved the necessary statistical reporting required by a screening program.

### P57. Are Low Birth Weight Children Predisposed to Renal Loss of Carnitine?

ProtasPiotr Tomasz^1^KępkaAlina^2^Rybi-SzuminskaAgnieszka^1^Taranta-JanuszKatarzyna^1^WasilewskaAnna^1^1Department of Pediatrics and Nephrology, Medical University of Białystok, 15-089 Białystok, Poland2Department of Biochemistry, Radioimmunology and Experimental Medicine, The Children’s Memorial Health Institute, 04-730 Warsaw, Poland

The plasma homeostasis of both free and esterified carnitines is mostly regulated by renal tubular reabsorption, which may be disturbed in low birth weight children. The aim of study was to check whether disturbances in L-carnitine [LC] C and its main ester, acetyl-carnitine [ALC] excretion, may be the result of renal dysfunction in low birth weight children (LBW). The study included 59 LBW children (2165 g [1490–2440]) and 22 children with normal birth weight as a reference group (3500 g [3275–3650]). Subjects were divided into three groups: 0–3 month, 4–12 month and over 1 year at the time of testing. Carnitine levels were measured spectrophotometrically. The urine excretion of Free LC, Free LC/cr., Total LC and Total LC/cr. were significantly higher in 0–3 and 4–12-month old LBW infants study groups when compared to the reference groups. We found statistically significant higher urine excretion of ALC and ALC/cr. in all age groups of LBW infants compared to the reference group. There was a negative correlation between birth weight and Free LC/cr. (r = −0.3, *p* < 0.05), Total LC/cr. (r = −0.34, *p* < 0.05), and ALC/cr. (r = −39, *p* < 0.05), and in the children >12-month old strong negative correlation between eGFR and free LC/cr. (r = −0.6, *p* < 0.05), Total LC/cr. (r = −0.61, *p* < 0.05), ALC/cr. (r = −0.61, *p* < 0.05). Higher urine excretion of both LC and ALC and its negative correlation with birth weight and eGFR may reflect some degree of renal dysfunction in LBW infants.

### P58. Newborn Screening for Lysosomal Storage Diseases: Metrics for Comparing Assay Platforms

GelbMichael H.RobinsonBruce H.Department of Chemistry, Univ. of Washington, Seattle, WA 98195, USA

All newborn screening (NBS) programs for lysosomal storage diseases (LSDs) measure the activity of lysosomal enzymes in dried blood spots. In our presentation we clarify points of confusion in the literature concerning LSD NBS metrics and results of live NBS programs. The Analytical Range and the Z-value have been suggested as useful metrics to evaluate LSD NBS assays. Here we show by rigorous mathematical analysis that it is the imprecision at the screen cutoff that is the most important metric for determining the rate of false positives. Data published by the CDC show that the imprecision at the screen cutoff using tandem mass spectrometry (MS/MS) is much lower than that using digital microfluidics fluorimetry (DMF-F) (https://www.cdc.gov/labstandards/nsqap_resources.html). This quantitatively explains the 2-to 4-fold lower false positive rates reported by worldwide live LSD NBS programs using MS/MS versus DMF-F. We also show that the positive predictive value (PPV) is not a useful metric at this stage of LSD NBS. There is discussion on use of multiple-of-the-median (MOM) so that universal cutoffs can be established across different NBS programs using different assay platforms. We show that this is not feasible for some LSDs including Pompe disease because the DMF-F assay shows much stronger interference from the off-target enzyme, maltase glucoamylase, than the MS/MS assay. We also show that the MS/MS LSD NBS assay using a single buffer to assay multiple enzymes gives superior analytical metrics than does the DMF-F method in which each enzyme is assayed its own buffer. It has been suggested that the substrate concentration used to assay lysosomal enzymes be above the Michaelis constant (KM) for the enzyme of interest otherwise the product versus time curve may be non-linear. There is no basis for the concern in the present case since only a tiny fraction of substrate is converted to substrate. All MS/MS and DMF-F product-versus-time curves are linear over the incubation period.

### P59. Tandem Mass Spectrometry for Newborn Screening of 15 Lysosomal Storage Diseases and Cerebrotendinous Xanthomatosis. Enormous Platform Flexibility and Results of Pilot Studies

GelbMichael H.^1^HongXinying^1^YiFan^1^KumarArun Babu^1^DaikerJessica^1^GhomashchiFarideh^1^PendemNagendar^1^MasiSophia^1^ScottC Ronald^2^1Department of Chemistry, Univ. of Washington, Seattle, WA 98195, USA2Department of Pediatrics, Univ. of Washington, Seattle, WA 98195, USA

Tandem mass spectrometry (MS/MS) has emerged as the major method for newborn screening (NBS) of lysosomal storage diseases (LSDs). The original panel of LSDs has been expanded to include MPS-II, MPS-IIIB, MPS-IVA, MPS-VI, and MPS-VII. Most recently we have added MPS-IIIA, lysosomal acid lipase, neuronal ceroid lipofuscinosis types 1 and 2, and metachromatic leukodystrophy (MLD). We now show how all of these diseases can be screened using a single UPLC-MS/MS run (2.1 min per sample). In addition to measuring the enzymatic activities by UPLC-MS/MS, we also can include quantification of several biomarker (sulfatides for MLD, lyso-sphingomyelin for Niemann-Pick-A/B, lyso-Gb3 for Fabry, bile alcohols for CTX, and C26-LPC for X-linked adrenoleukodystrophy). Of special note, we report the first assay for MPS-IIIA using a dried blood spot sample; previous assays were possible only with more plentiful sample (i.e., fibroblasts). Our lysosomal acid lipase substrate is specific so that multiple assays with and without enzyme inhibitor are no longer needed. The UPLC-MS/MS is thus very flexible, and it can also be expanded to include additional disorders. We have completed a pilot study (~100,000 newborns) in the WA NBS lab of MPS-II, MPS-IIIB, MPS-IVA, MPS-VI, and MPS-VII. The number of false positives is remarkably low (<10 false positives per disease per 100,000 newborns). Confirmed affected patients were also identified (based on essentially zero enzymatic activity combined with severe mutations or by clinical examination). NBS for these disorders is thus suggested to be feasible by the LC-MS/MS method. We are also conducting a pilot study for NBS of MLD and CTX, with data to ~60,000 now available. The number of false positives is sufficiently small that we can now be confident that NBS for these additional disorders by LC-MS/MS will be feasible.

